# Pharmacokinetic–pharmacodynamic guided optimisation of dose and schedule of CGM097, an HDM2 inhibitor, in preclinical and clinical studies

**DOI:** 10.1038/s41416-021-01444-4

**Published:** 2021-06-17

**Authors:** Sebastian Bauer, George D. Demetri, Ensar Halilovic, Reinhard Dummer, Christophe Meille, Daniel S. W. Tan, Nelson Guerreiro, Astrid Jullion, Stephane Ferretti, Sebastien Jeay, Laurence Van Bree, Florence Hourcade-Potelleret, Jens U. Wuerthner, Claire Fabre, Philippe A. Cassier

**Affiliations:** 1 Department of Medical Oncology, Sarcoma Center, West German Cancer Center, University of Duisburg-Essen, Duisburg-Essen, Germany; 2 Dana-Farber Cancer Institute and Ludwig Center at Harvard Medical School, Boston, MA USA; 3 Novartis Institutes for BioMedical Research (NIBR), Cambridge, MA USA; 4 University Hospital Zurich, Zurich, Switzerland; 5 Novartis Institutes for BioMedical Research (NIBR), Basel, Switzerland; 6 National Cancer Center Singapore, Singapore, Singapore; 7 Department of Medical Oncology, Centre Léon Bérard, Lyon, France; 8 Present Address: F. Hoffmann-La Roche AG, Basel, Switzerland; 9 Present Address: Idorsia Pharmaceuticals Ltd, Allschwil, Switzerland; 10 Present Address: ADC Therapeutics, Epalinges, Switzerland

**Keywords:** Drug discovery, Drug development

## Abstract

**Background:**

CGM097 inhibits the p53-HDM2 interaction leading to downstream p53 activation. Preclinical in vivo studies support clinical exploration while providing preliminary evidence for dosing regimens. This first-in-human phase I study aimed at assessing the safety, MTD, PK/PD and preliminary antitumor activity of CGM097 in advanced solid tumour patients (NCT01760525).

**Methods:**

Fifty-one patients received oral treatment with CGM097 10–400 mg 3qw (*n* = 31) or 300–700 mg 3qw 2 weeks on/1 week off (*n* = 20). Choice of dose regimen was guided by PD biomarkers, and quantitative models describing the effect of CGM097 on circulating platelet and PD kinetics.

**Results:**

No dose-limiting toxicities were reported in any regimens. The most common treatment-related grade 3/4 AEs were haematologic events. PK/PD models well described the time course of platelet and serum GDF-15 changes, providing a tool to predict response to CGM097 for dose-limiting thrombocytopenia and GDF-15 biomarker. The disease control rate was 39%, including one partial response and 19 patients in stable disease. Twenty patients had a cumulative treatment duration of >16 weeks, with eight patients on treatment for >32 weeks. The MTD was not determined.

**Conclusions:**

Despite delayed-onset thrombocytopenia frequently observed, the tolerability of CGM097 appears manageable. This study provided insights on dosing optimisation for next-generation HDM2 inhibitors.

**Translational relevance:**

Haematologic toxicity with delayed thrombocytopenia is a well-known on-target effect of HDM2 inhibitors. Here we have developed a PK/PD guided approach to optimise the dose and schedule of CGM097, a novel HDM2 inhibitor, using exposure, platelets and GDF-15, a known p53 downstream target to predict patients at higher risk to develop thrombocytopenia. While CGM097 had shown limited activity, with disease control rate of 39% and only one patient in partial response, the preliminary data from the first-in-human escalation study together with the PK/PD modeling provide important insights on how to optimize dosing of next generation HDM2 inhibitors to mitigate hematologic toxicity.

## Introduction

Tumour suppressor p53 is a transcription factor that controls the expression of a myriad of target genes involved in DNA repair, apoptosis, and cell-cycle arrest, which are all important processes counteracting the malignant growth of tumours. TP53 is one of the most commonly mutated genes in human cancer. Approximately 50% of all human cancers harbour TP53 mutations. In cancers where the TP53 gene is not mutated, the function of the p53 pathway is often suppressed through mechanisms that affect the stability and activity of the p53 protein. One such mechanism is overexpression or deregulation of MDM2. MDM2, for which the human orthologue is also known as HDM2, is an E3 ubiquitin ligase, which by direct binding negatively regulates p53 through ubiquitination and subsequent proteasomal degradation.^1^

CGM097 inhibits the p53-HDM2 interaction which protects p53 from degradation, subsequently leading to its accumulation and activation resulting in stimulation of downstream effector pathways that induce cell-cycle arrest and/or, apoptosis through transcriptional activation of cell-cycle inhibitory genes (e.g. CDKN1A), and pro-apoptotic genes (e.g. PUMA and NOXA).^2–4^ HDM201 inhibits the growth, in vitro and in vivo, of tumour models with functional wild-type p53 derived from a variety of cancer types.^2–5^ Here, we present the preclinical validation and the results of the first-in-human phase I study conducted to determine the safety, maximum tolerated dose (MTD), pharmacokinetic/pharmacodynamic (PK/PD), and preliminary antitumor activity of CGM097 in patients with advanced solid tumours. In addition, we describe the development of an integrated PK/PD modelling approach to characterise the temporal dose–response relationships of drug action on platelets and serum growth differentiation factor-15 (GDF-15) as a downstream marker of p53 pathway activation; predict platelet response and incidence rates of thrombocytopenia; and identify associations between GDF-15 induction and risk of developing delayed thrombocytopenia. Several tumours with high a priori p53 wild-type status, including melanoma, soft-tissue sarcoma, osteosarcoma, renal cell carcinoma, and proximal colorectal tumours, were investigated.^6–10^

## Methods

### Preclinical studies

#### Animal study design

All animal experiments were performed according to procedures covered by permit number BS-1975 issued by the Cantonal Veterinary Office, Basel, Switzerland, and in strict adherence to the Federal Animal Protection Act and the Federal Animal Protection Code. Female athymic nude mice (Hsd:Athymic Nude-Foxn1nu, 7–8 weeks old, 20–22 g) were obtained from Envigo (Germany). All animals were housed in a pathogen-controlled environment with access to food and water ad libitum, and were identified with transponders.

CGM097 was freshly dissolved in 0.5% hydroxypropyl methylcellulose for PO administration at 10 mL/kg. For PK/PD experiments, animals were randomised into groups of three, and tissue samples were collected at 0, 1, 3, 8, 16, 24 and 48 h. At the times indicated, animals were anaesthetised by exposure to 2–3% v/v isoflurane in medical oxygen. For efficacy experiments, animals were randomised into groups (*n* = 6) for a mean tumour size of 100 mm^3^, and CGM097 was administered at several doses according to the respective dosing regimen for 14 days.

#### Assessments

Subcutaneous SJSA-1, 778 and LP6 tumours were induced by injecting tumour cells expanded in vitro into the right flank of nude mice. For (PK)/(PD) experiments, CGM097 was injected once at 50 or 100 mg/kg. After collecting blood samples for determining plasma concentrations of CGM097, the animals were sacrificed before they recovered from anaesthesia. The tumours were excised, weighed and rapidly frozen in liquid nitrogen. Later, they were cryogenically dry pulverised using the CryoPrep™ system (model CP-02, Covaris).

Tumour response was reported with the measures of tumour volumes from the treatment start. Concentrations of CGM097 in plasma were determined simultaneously by ultra-performance liquid chromatography–tandem mass spectrometry. The total RNA was purified from cell pellets using the QIAshredder (cat # 79654, Qiagen) and RNeasy Mini Kit (74106, Qiagen) according to the manufacturer’s instructions, with the exception that no DNA digestion was performed. Total RNA was quantitated using the spectrophotometer ND-1000 Nanodrop^®^. Quantitative reverse transcriptase PCR (qRT-PCR) was set up in triplicate per sample using either the One-Step RT qPCR Master Mix Plus (RT-QPRT-032X, Eurogentec) or the iTaq™ Universal Probes One-Step Kit (#1725141), with primers from TaqMan Gene Expression assays (20 × probe dye FAM™ [or VIC]-TAMRA [or MGB]; Applied Biosystems), including control primers (*GUS*-β 4310888E-1012026) and primers for the target genes (CDKN1A Hs00355782_m1; BBC3 Hs00248075_m1; MDM2 Hs01066930_m1). Alternatively, we performed a multiplex gene expression analysis on 104 p53 target genes with the NanoString assay.

### Clinical study

#### Study design

This phase I, multicenter, open-label study (NCT01760525) evaluated CGM097 as an oral single-agent administered in a continuous three times a week (3qw) schedule in patients with advanced solid tumours (regimen 1). The protocol allowed switching to one or more of three alternative regimens; of these, the intermittent dosing regimen of 3qw for 2 weeks on/1 week off was investigated. The study was conducted in accordance with the Declaration of Helsinki and Good Clinical Practice and approved by Institutional review boards. All patients provided written informed consent.

#### Patients

Eligible patients were ≥18 years old, had locally advanced or metastatic solid tumours (including but not restricted to melanoma, proximal colorectal cancer, soft-tissue sarcoma, osteosarcoma or renal cell carcinoma) with evidence of disease progression, and had failed or been ineligible for standard-of-care therapy with a World Health Organisation performance status of 0 to 2 and adequate organ function. All patients were required to have TP53^*w*t^ status or, at a minimum, no mutations in exons 5, 6, 7 and 8 determined locally whenever applicable and/or assessed centrally. TP53 status could be determined on archival samples no older than 36 months at study entry. Patients having a prior treatment with compounds with the same mechanism of action as CGM097 were excluded. Patients with central nervous system metastatic lesions or concurrent other malignancy or clinically significant cardiac disease or abnormal laboratory findings were excluded.

#### Assessments

The primary objective was to determine the MTD and/or identify the recommended dose for expansion of CGM097 for the daily and intermittent dosing regimens.

Safety was assessed according to the National Cancer Institute Common Terminology Criteria for Adverse Events (NCI-CTCAE) v4.03. Antitumor activity was assessed by Response Evaluation Criteria In Solid Tumours (RECIST) v1.1. Serial blood samples were collected throughout cycle 1, then sparse samples on day 1 of cycle 2 and predose samples on subsequent cycles, to determine plasma CGM097 concentrations. Fresh biopsies were collected on day 8 of cycle 1 along with a matching PK sample (6 h ± 2 h post-dose). Plasma concentrations of CGM097 were measured using a validated liquid chromatography–tandem mass spectrophotometry approach, with a lower limit of quantification of ~1 ng/mL.

### Pharmacodynamic tumour marker evaluation

Serum growth differentiation factor-15 (GDF-15) was measured using the Quantikine ELISA kit (#DGD150, R&D Systems). Briefly, serum samples were diluted 1:4 and transferred to pre-coated plates. The assay was run with an 8-point standard curve, and absorbance was read at 450 nM. All samples were assayed in duplicate, and mean values were reported if within range (25–45,000 pg/mL). A coefficient of variation (CV%) < 20 between duplicates was considered acceptable. The fold increase in GDF-15 at 24 h post-dose CGM097 (cycle 1, day 2) was measured relative to baseline.

### Statistical analysis

A Bayesian Logistic Regression Method (BLRM) employing the Escalation With Overdose Control (EWOC) was used during the escalation phase for the selection of doses. Determination of the MTD during escalation was based upon the estimation of the probability of DLT in cycle 1 in patients belonging to the dose-determining set. Dose escalation decisions were based on a clinical synthesis of all relevant available data (toxicity, PK, and PD information) together with the DLT information.

Antitumor efficacy was assessed using computerised tomography (CT)/magnetic resonance imaging assessment at baseline and every 8 weeks until disease progression or the initiation of subsequent antineoplastic therapy or death, whichever occurred first. In addition, the criteria for determining confirmed partial response (PR) or complete response (CR) were required to be present for at least 4 weeks. Demographic and other baseline data and PK/PD parameters were summarised using descriptive statistics.^11^ PK parameters were estimated using noncompartmental methods (Phoenix^®^, Pharsight, Mountain View, California, USA).

### Pharmacokinetic and pharmacodynamic modelling

A total of 1919 observations (924 plasma PK, 717 platelet, and 278 GDF-15 measurements) and 20 platelet transfusion events derived from 46 patients were included in the dataset (data cutoff, January 2016). A nonlinear mixed-effect modelling approach^12^ was used to formulate the models describing the PK, serum GDF-15 and platelet time-course relationship with CGM097 treatment. We used the stochastic approximation of the expectation-maximisation (SAEM) algorithm^13^ implemented in the Monolix software (version 4.3.2, Lixoft) to estimate the fixed (population-level typical values) and random (inter-individual variability) effects for each model parameter and a residual error.

In brief, the PK properties of CGM097 was described by a 2-compartment model with a delayed first-order absorption and a linear elimination. A semi-mechanistic model mimicking hematopoietic cell maturation and regulation was developed to describe the time course of platelet kinetics and drug action on immature cells and modified from Friberg et al.^14^ The change in serum GDF-15 in response to CGM097 was described by a type III indirect response PD model with drug stimulation of GDF-15 production as previously described.^15,16^ A schematic diagram of the PK and PK/PD models for platelets (thrombocytopenia) and for GDF-15 time-course relationship is presented in Supplementary Fig. S1. The equations for the respective models are provided in the Supplementary Material (Supplementary Data).

Models were evaluated based on the change in the Akaike’s information criterion (AIC) value, the convergence of the SAEM algorithm, the precision of the parameter estimates (NLME derived % relative standard error [RSE%]) and decreases in IIV and residual variability. Diagnostic plots were used to evaluate model adequacy, including evaluation of observations versus population and individual predicted (IPRED), and the distributions of conditional weighted residuals (CWRES) or normalised prediction distribution error (NPDE) versus time/observations or over time plots (Supplementary Figs. S2–S4). The predictive performance of the final models was evaluated by simulating 500 datasets using parameter estimates (fixed and random effects) and plotting a visual predictive check (Supplementary Fig. S5). For serum GDF-15, serum concentration levels above the upper limit of quantification (>50,000 pg/mL) were handled as right-censored data.

## Results

### Identification of the three times a week (3qw) dosing regimen in preclinical, PK, PD, efficacy study

**Fig. 1:**
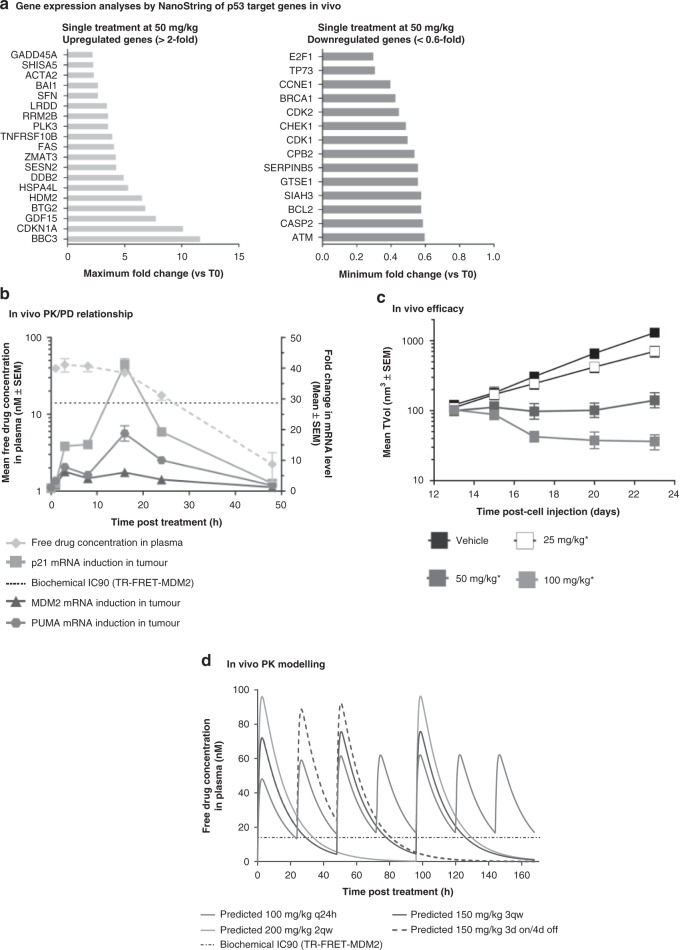
Preclinical data in SJSA-1 tumour-bearing mice treated with CGM097. **a** NanoString analysis with selected known p53-regulated genes performed in SJSA-1 tumours from animals treated with a single 50 mg/kg dose of CGM097. **b** For PK/PD experiment, mice were randomised into groups of *n* = 3 and sacrificed/sampled over a 48 h period of time. Unbound plasma exposure was calculated on the basis of a free fraction of 0.193% in the mouse. The black dotted lines represent the biochemical IC90. **c** For efficacy experiment, mice were randomised into groups of *n* = 6 and CGM097 was administered at several doses according to respective dosing regimen for 14 days. Differences between the means of TVol were assessed on the endpoint using a one-way ANOVA with Dunnett’s tests post hoc (*P < 0.05 considered as significant). **d** One-compartment PK model (WinNonlin/Phoenix 6.3) of the mouse was used to estimate PK parameters (average plasma concentrations at steady-state [C_ave-ss_] and maximum plasma concentrations at steady-state [C_max-ss_]) for the four dosing regimens and correlate with the observed regression. **a** Gene expression analyses by NanoString of p53 target genes in vivo. **b** In vivo PK/PD relationship. **c** In vivo efficacy. **d** In vivo PK modelling.

NanoString analysis with selected known p53-regulated genes was performed in SJSA-1 tumours from animals treated with a single 50 mg/kg dose of CGM097 to identify the most highly up- and downregulated p53 target genes. Results revealed the activation of p53 transcriptional function, with a 12-fold increase in BCL2-binding component 3 (BBC3), a pro-apoptotic member of the Bcl-2 protein family, also known as p53-upregulated modulator of apoptosis (PUMA). Cyclin-dependent kinase inhibitor 1 (CDKN1A), also known as p21, was upregulated by a factor of 10. GDF-15, also known as MIC1, and HDM2 were upregulated, albeit to a lesser extent. On the other hand, the activation of p53 caused a significant decrease in mRNA expression of E2F1 and TP73, while there was no effect on TP63 expression. Genes involved in cell-cycle regulation (CDK1, CDK2, CCNE1, GTSE1) and DNA repair (ATM, BRCA1, CHEK1), and BCL-2 were also downregulated (Fig. 1a).

To characterise the PK/PD relationship, we applied the theory of “free-drug hypothesis”, which states that the pharmacological activity of a drug is determined by the unbound drug concentration. The estimated unbound fractions in plasma were 0.193% ± 0.0567% for the mouse. Treatment with CGM097 at 100 mg/kg in SJSA-1 tumour-bearing mice showed that the unbound drug concentration in plasma (plasma protein binding = 99.8% in mouse) stayed above the in vitro biochemical inhibitory concentration 90% (IC90) (14 nM) for the first 24 h^17^ and such sustained exposure highly induced p21 (maximum effect Emax = 41-fold) and PUMA, (Emax = 19-fold) (Fig. 1b). HDM2 had the lowest increase in expression among these three genes. As previously published,^18^ the time to reach the Emax is delayed compared to plasma Cmax as p53 target genes accumulate as long as unbound drug concentration in plasma stay above the threshold and p53 remains activated. Once the unbound drug concentration decreased below the IC_90_, the pharmacological activity dramatically decreased and reached basal levels 48 h post treatment (Fig. 1b).

Daily treatment of tumour-bearing mice with CGM097 dose-dependently affected SJSA-1 tumour growth in vivo (Fig. 1c). When dosed daily at 100 mg/kg, 65% of tumour regression was reached after 10 days of treatment. When dosed daily at 50 mg/kg, stable disease (SD) was observed (T/C = 3%); tumour growth was reduced by 50% at the 25 mg/kg dose compared to the vehicle treatment group. Similar tumour growth inhibition and dose–response was reached in additional MDM2-amplified well-differentiated (WDLPS) and dedifferentiated liposarcoma (DDLPS) models (Supplementary Fig. S6). Three additional dosing regimens (3qw, 2qw or 3 days on/4 days off) were also investigated, and similar regression levels were observed in comparison with the daily dosing regimen (Fig. 1d), confirming that the efficacy of CGM097 is not dosing regimen dependent. Based on a one-compartment PK model (WinNonlin/Phoenix 6.3) of mouse, PK parameters (average plasma concentrations at steady-state [C_ave-ss_] and maximum plasma concentrations at steady-state [C_max-ss_]) were simulated for the three alternative dosing regimens in addition to the daily regimen (Fig. 1d). Among all the dosing regimens inducing similar tumour regression, the 3qw regimen was expected to result in the best therapeutic index in the clinic, as it had a lower weekly dose (450 mg/kg) and C_ave-ss_ (27.9 nM) compared with the standard daily treatment regimen (700 mg/kg and 37.3 nM), and a lower C_max-ss_ (75.8 nM) compared with the 2qw (96.4 nM) and 3 days on/4 days off (92.3 nM) regimens.

### Treatment regimens and study population

In the clinical study, 51 patients with advanced solid tumours were enrolled in five centres across five countries. In regimen 1, 31 patients were administered CGM097 3qw continuously until disease progression at doses of 10 mg (*n* = 3), 20 mg (*n* = 4), 40 mg (*n* = 4), 80 mg (*n* = 4), 150 mg (*n* = 4), 300 mg (*n* = 7) and 400 mg (*n* = 5). Twenty patients were treated with the alternative regimen, 2 weeks on/1 week off at doses of 300 mg (*n* = 8), 500 mg (*n* = 6) and 700 mg (*n* = 6). Twenty patients (39.2%) had a cumulative treatment duration of >16 weeks, with eight patients (15.7%) reporting more than 32 weeks of treatment.

At the cutoff date (July 7, 2016), the median treatment duration for all patients was 9.7 weeks (1.7–118 weeks). All except four patients, received more than 4 weeks of treatment, with four patients (7.8%) reporting 24–32 weeks and eight patients (15.7%) reporting more than 32 weeks of treatment, respectively. The mean treatment duration was the longest in the CGM097 (400 mg) regimen 1 treatment group (36.1 weeks). Demographics and patient disposition are summarised (Table 1).

**Table 1:** Demographics and patient disposition by treatment groups.

Demographic variables	10 mg Reg 1, *N* = 3	20 mg Reg 1, *N* = 4	40 mg Reg 1, *N* = 4	80 mg Reg 1, *N* = 4	150 mg Reg 1, *N* = 4	300 mg Reg 1, *N* = 7	400 mg Reg 1, *N* = 5	300 mg Reg 3, *N* = 8	500 mg Reg 3, *N* = 6	700 mg Reg 3, *N* = 6	All patients, *N* = 51
Age (years)											
Mean (SD)	47 (8.89)	50 (9.13)	50.8 (13.65)	59.3 (5.38)	44.5 (7.0)	58.7 (7.67)	56.8 (12.05)	50.3 (12.51)	56.3 (11.55)	56.7 (8.26)	53.6 (10.33)
Age group (years), *n* (%)											
<65	3 (100)	4 (100)	3 (75.0)	3 (75.0)	4 (100)	5 (71.4)	3 (60.0)	7 (87.5)	5 (83.3)	5 (83.3)	42 (82.4)
≥65	0	0	1 (25.0)	1 (25.0)	0	2 (28.6)	2 (40.0)	1 (12.5)	1 (16.7)	1 (16.7)	9 (17.6)
Sex, *n* *(%)*											
Male	1 (33.3)	3 (75.0)	3 (75.0)	2 (50.0)	1 (25.0)	2 (28.6)	2 (40.0)	3 (37.5)	2 (33.3)	3 (50.0)	22 (43.1)
Female	2 (66.7)	1 (25.0)	1 (25.0)	2 (50.0)	3 (75.0)	5 (71.4)	3 (60.0)	5 (62.5)	4 (66.7)	3 (50.0)	29 (56.9)
WHO PS, *n* (%)											
0	2 (66.7)	3 (75.0)	3 (75.0)	2 (50.0)	2 (50.0)	4 (57.1)	4 (80.0)	4 (50.0)	3 (50.0)	4 (66.7)	31 (60.8)
1	0	1 (25.0)	0	1 (25.0)	2 (50.0)	2 (28.6)	1 (20.0)	4 (50.0)	3 (50.0)	2 (33.3)	16 (31.4)
2	1 (33.3)	0	1 (25.0)	1 (25.0)	0	1 (14.3)	0	0	0	0	4 (7.8)
Patient disposition, *n* (%)											
Treatment discontinued	3 (100)	4 (100)	4 (100)	4 (100)	4 (100)	6 (85.7)	4 (80.0)	8 (100)	6 (100)	6 (100)	49 (96.1)
Treatment ongoing	0	0	0	0	0	1 (14.3)	1 (20.0)	0	0	0	2 (3.9)
Primary reason for treatment end, *n* (%)											
Adverse event	0	0	0	0	0	1 (14.3)	2 (40.0)	1 (12.5)	0	1 (16.7)	5 (9.8)
Consent withdrawn	0	0	0	0	0	0	0	0	0	1 (16.7)	1 (2.0)
Death	1 (33.3)	0	1 (25.0)	0	0	0	0	0	0	0	2 (3.9)
Disease progression	2 (66.7)	4 (100)	3 (75.0)	4 (100)	4 (100)	5 (71.4)	2 (40.0)	7 (87.5)	6 (100)	4 (66.7)	41 (80.4)

*Reg* regimen, *PS* performance status.

Reg 1: 3qw dosing, 4-week treatment cycle, continuous; Reg 3: 3qw dosing, 3-week treatment cycle; 2 weeks on treatment and 1 week off treatment.

### Clinical safety

No formal DLTs were observed during the study. However, DLT-like events reported after day 28 (cycle 1) suggested that continuous 3qw dosing was not well-tolerated, leading to delayed grade 3 or 4 thrombocytopenia. The onset of on-target myelosuppression appeared to be delayed, however, the effect was cumulative over time. Twenty-five patients experienced grade 3 AEs and 12 patients experienced grade 4 AEs regardless of study drug relationship. Most frequently reported AEs (any grade) were nausea (*n* = 24), thrombocytopenia (*n* = 22), anaemia, fatigue and vomiting (*n* = 18 patients each). Grade 3 AEs suspected to be study drug-related were observed in 11 patients, and 10 patients reported at least one grade 4 AE. The incidence of grade 3/4 AEs suspected to be drug-related was more common at doses ≥300 mg. In addition, the incidence of delayed haematologic AEs generally increased with increasing dose on the continuous dosing regimen (3qw); however, the incidence was reduced on the alternative dosing regimen (2 weeks on/1 week off), despite higher doses of treatment.

The most common treatment-related grade 3 or 4 AEs were thrombocytopenia (*n* = 12), lymphopenia (*n* = 6), and neutropenia (*n* = 6) (Table 2). The most frequently reported treatment-related AEs included thrombocytopenia (*n* = 22), nausea (*n* = 21), leukopenia (*n* = 14), vomiting (*n* = 13) and fatigue (*n* = 13) (Table 2). Overall, five patients had at least one AE leading to study drug discontinuation. Seven deaths occurred on treatment, two while receiving the study drug and five within 30 days of treatment discontinuation; none were suspected to be treatment-related.

**Table 2:** (A) Grade 3 or 4 adverse events suspected to be related to study drug; (B) adverse events suspected to be related to study drug (all grades, in ≥ 10% of patients).

	10 mg Reg 1	20 mgReg 1	40 mgReg 1	80 mgReg 1	150 mg Reg 1	300 mgReg 1	400 mgReg 1	300 mgReg 3	500 mgReg 3	700 mgReg 3	All patients
	*N* = 3	*N* = 4	*N* = 4	*N* = 4	*N* = 4	*N* = 7	*N* = 5	*N* = 8	*N* = 6	*N* = 6	*N* = 51
(A) Preferred term, *n* %											
Thrombocytopenia	0	0	0	0	0	4 (57.1)	4 (80.0)	0	1 (16.7)	3 (50.0)	12 (23.5)
Lymphopenia	1 (33.3)	0	0	0	0	0	1 (20.0)	1 (12.5)	1 (16.7)	1 (16.7)	5 (9.8)
Neutropenia	0	0	0	0	0	1 (14.3)	1 (20.0)	0	1 (16.7)	3 (50.0)	6 (11.8)
Lipase increased	0	0	1 (25.0)	0	0	1 (14.3)	0	1 (12.5)	1 (16.7)	1 (16.7)	5 (9.8)
Leukopenia	0	0	0	0	0	1 (14.3)	2 (40.0)	0	0	1 (16.7)	4 (7.8)
Anaemia	0	0	0	0	0	2 (28.6)	0	0	0	2 (33.3)	4 (7.8)
White blood cell count decreased	0	0	0	0	0	3 (42.9)	1 (20.0)	0	0	0	4 (7.8)
Granulocytopenia	0	0	0	0	0	1 (14.3)	0	0	0	0	1 (2.0)
Nausea	0	0	0	0	0	0	0	1 (12.5)	1 (16.7)	0	2 (3.9)
Lymphocyte count decreased	0	0	0	0	0	1 (14.3)	0	0	0	0	1 (2.0)
Neutrophil count decreased	0	0	0	0	0	2 (28.6)	0	0	0	0	2 (3.9)
Hyperlipasaemia	0	0	0	0	0	0	0	0	0	1 (16.7)	1 (2.0)
Hyperlipidaemia	0	0	0	0	0	1 (14.3)	0	0	0	0	1 (2.0)
Proteinuria	0	0	0	0	0	0	1 (20.0)	0	0	0	1 (2.0)

(B) Preferred term, *n* %											
Thrombocytopenia	0	0	0	0	1 (25.0)	6 (85.7)	5 (100.0)	2 (25.0)	4 (66.6)	4 (66.6)	22 (43)
Nausea	0	0	0	0	0	5 (71.4)	3 (60.0)	5 (62.5)	5 (83.3)	4 (66.6)	22 (43)
Leukopenia	1 (33.3)	0	1 (25.0)	0	0	3 (42.8)	4 (80.0)	1 (12.5)	1 (16.7)	3 (50.0)	14 (27)
Fatigue	1 (33.3)	0	1 (25.0)	0	0	4 (57.1)	1 (20.0)	3 (37.5)	2 (33.3)	1 (16.7)	13 (26)
Vomiting	0	1 (25.0)	0	0	0	2 (28.6)	3 (60.0)	3 (37.5)	3 (50.0)	2 (33.3)	14 (27)
Diarrhoea	0	1 (25.0)	0	0	1 (25.0)	2 (28.6)	2 (40.0)	2 (25.0)	1 (16.7)	2 (33.3)	11 (22)
Neutropenia	0	0	0	0	0	3 (42.8)	3 (60.0)	1 (12.5)	1 (16.7)	3 (50.0)	11 (22)
Lymphopenia	1 (33.3)	0	1 (25.0)	0	1 (25.0)	1 (14.3)	1 (20.0)	2 (25.0)	2 (33.3)	1 (16.7)	10 (20)
Decreased appetite	0	0	0	0	0	3 (42.8)	2 (40.0)	1 (12.5)	1 (16.7)	2 (33.3)	9 (18)
Anaemia	0	0	0	1 (25.0)	0	2 (28.6)	3 (60.0)	0	0	2 (33.3)	8 (16)
Dysgeusia	0	0	1 (25.0)	0	0	0	0	2 (25.0)	2 (33.3)	2 (33.3)	7 (14)
Abdominal pain upper	0	0	0	0	0	1 (14.3)	1 (20.0)	1 (12.5)	2 (33.3)	1 (16.7)	6 (12)
Asthenia	1 (33.3)	1 (25.0)	0	0	1 (25.0)	1 (14.3)	1 (20.0)	0	0	2 (33.3)	7 (14)
Blood CPK increased	0	0	1 (25.0)	1 (25.0)	0	2 (28.6)	0	2 (25.0)	0	0	6 (12)
Lipase increased	0	0	1 (25.0)	0	0	1 (14.3)	1 (20.0)	1 (12.5)	1 (16.7)	1 (16.7)	6 (12)
ALT increased	0	2 (50.0)	0	0	0	1 (14.3)	1 (20.0)	0	0	1 (16.7)	5 (10)
AST increased	0	1 (25.0)	0	0	1 (25.0)	1 (14.3)	1 (20.0)	1 (12.5)	0	0	5 (10)
Dizziness	0	1 (25.0)	0	0	0	1 (14.3)	1 (20.0)	1 (12.5)	1 (16.7)	0	5 (10)
Hypoalbuminemia	0	0	0	0	2 (50.0)	1 (14.3)	1 (20.0)	0	0	1 (16.7)	5 (10)

*ALT* alanine aminotransferase, *AST* aspartate amino transferase, *CPK* creatine phosphokinase.

Adverse events suspected to be related to study drug (all grades, in ≥10% of patients.

Reg 1: 3qw dosing, 4-week treatment cycle, continuous; Reg 3: 3qw dosing, 3-week treatment cycle; 2 weeks on treatment and 1 week off treatment.

Because of the expected haematological on-target toxicity of MDM2 inhibitors, haematologic AEs were identified as adverse events of special interest (AESI) and included neutropenia, thrombocytopenia, leucopenia, anaemia and lymphopenia.^19^ AESIs occurred in 64.7% of patients regardless of study drug relationship, and in 51% as suspected to be treatment-related. Thrombocytopenia was the most common AESI (22 patients, 43.1%); all being suspected to be treatment-related; occurred more frequently at higher doses and was present in all patients on the 400 mg dose in regimen 1. Anaemia was the next most common AESI regardless of relationship; however, the least common drug-related AESI, with no obvious relationship to dose. Neutropenia occurred more frequently at higher doses, particularly with the 300 mg and 400 mg doses in the continuous 3qw dosing regimen; nearly all were considered to be treatment-related.

### Clinical efficacy

**Fig. 2:**
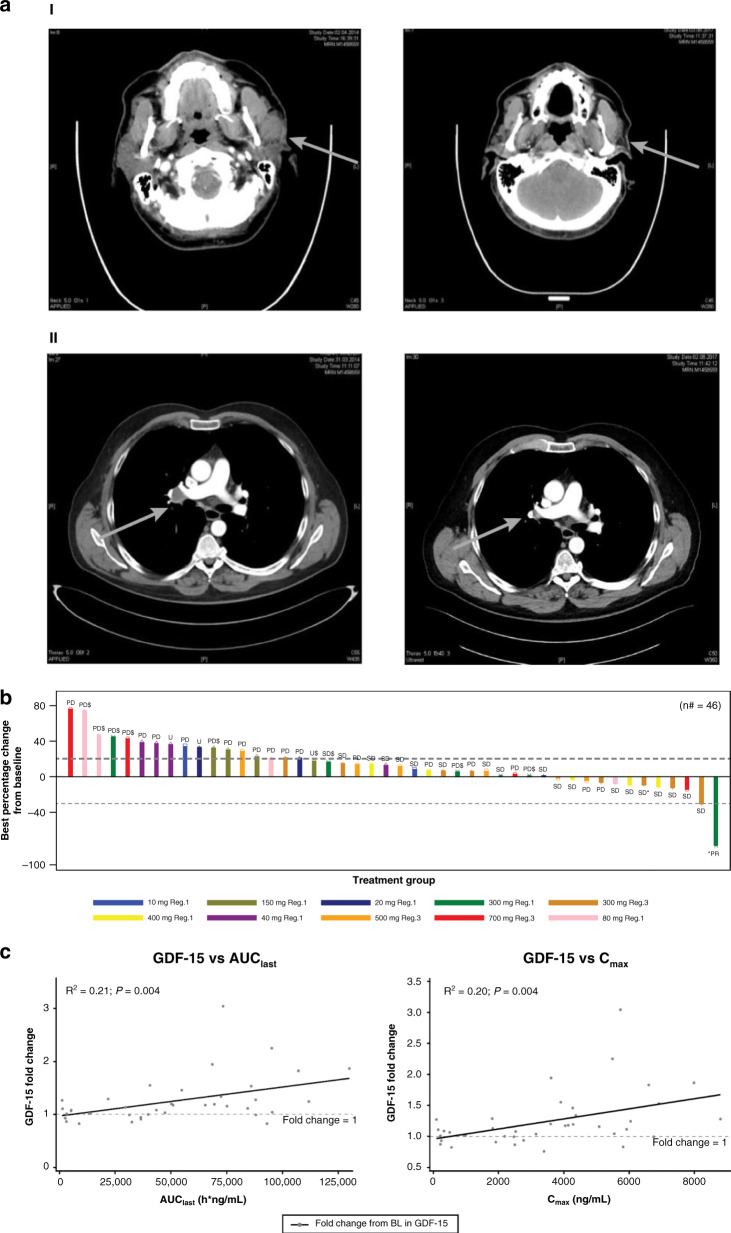
Clinical efficacy and pharmacokinetic/pharmacodynamic data. **a** (I) Reduction of tumour size (brain CT) in a malignant skin melanoma patient treated with CGM097 (300 mg 3 qw) observing partial clinical response (left before, right after treatment). (II) Reduction of tumour size (thorax CT) in a malignant skin melanoma patient treated with CGM097 (300 mg 3 qw) observing partial clinical response (left before, right after treatment). **b** Best-percentage change from baseline in the sum of longest diameters with local mutational status and treatment group (full analysis set). *n* = 46 is the number of patients for which the change in the total sum of the longest lesion diameters could be calculated, and best overall response as per investigators. Patients with baseline measurement only are not presented. Patients without any complete post baseline assessments are not included. Patients for which baseline and complete last assessments were available are included, even if these patients had missing assessments between baseline and last tumor assessments (U: unknown response, as per investigator assessments and in line with RECIST evaluation criteria). An asterisk symbol shows patients ongoing at the time of cut-off date. Symbol “$” shows patients having P53 mutation. n# number of subjects with baseline and post baseline CT scan results available. Reg. 1: 3 times weekly dosing, 4 weeks treatment cycle, continuous; Reg. 3: 3 times weekly dosing, 3 weeks treatment cycle; 2 weeks treatment, 1 week off treatment. **c** GDF-15-fold change from baseline versus AUC_last_ and C_max_. Each point represents an individual patient’s exposure and GDF-15 fold change. The continuous line represents linear regression with associated R^2^ and *P* value. The dots are individual data, the full line displays a linear regression model through the data, with *R*-square indicating the proportion of the variability in the dependent variable (GDF-15) that is explained by the model, and *P* value determining the statistical significance of the model. AUC_last_ area under the curve from time = 0 to last measurable concentration, BL baseline, C_max_ maximum concentration, GDF-15 growth differentiation factor 15.

Fifty-one patients were evaluated for efficacy every 8 weeks and/or at the end of treatment with CGM097. Nineteen patients, ten on the continuous and nine on the alternative dosing regimen, had SD as the best response. One patient with malignant melanoma on CGM097 300 mg (3qw dosing continuous) had PR (Fig. 2a) and the response was ongoing after 118 weeks at the time of cutoff date. Clinical benefit was observed in 20 patients; the disease control rate was 39% (20/51 patients). No CR occurred at any dose (Table 3). The correlation between efficacy and the average CGM097 dose intensity by cycle is represented in Supplementary Fig. S7. This figure illustrates that the majority of the clinical SD (and PR) were observed in the dose intensity ranges predicted to be active in preclinical efficacy studies.

**Table 3:** Summary of BOR per RECIST as per investigator assessment by treatment groups (full analysis set).

	10 mg Reg 1, *N* = 3	20 mg Reg 1, *N* = 4	40 mg Reg 1, *N* = 4	80 mg Reg 1, *N* = 4	150 mg Reg 1, *N* = 4	300 mg Reg 1, *N* = 7	400 mg Reg 1, *N* = 5	300 mg Reg 3, *N* = 8	500 mg Reg 3, *N* = 6	700 mg Reg 3, *N* = 6	All patients, *N* = 51
BOR											
CR	0	0	0	0	0	0	0	0	0	0	0
PR	0	0	0	0	0	1 (14.3)	0	0	0	0	1 (2.0)
SD	1 (33.3)	1 (25.0)	1 (25.0)	1 (25.0)	0	2 (28.6)	4 (80.0)	5 (62.5)	3 (50.0)	1 (16.7)	19 (37.3)
PD	2 (66.7)	2 (50.0)	2 (50.0)	3 (75.0)	3 (75.0)	3 (42.9)	1 (20.0)	3 (37.5)	3 (50.0)	3 (50.0)	25 (49.0)
Unknown	0	1 (25.0)	1 (25.0)	0	1 (25.0)	1 (14.3)	0	0	0	2 (33.3)	6 (11.8)
ORR	0	0	0	0	0	1 (14.3)	0	0	0	0	1 (2.0)
95% CI	[0.0, 70.8]	[0.0, 60.2]	[0.0, 60.2]	[0.0, 60.2]	[0.0, 60.2]	[0.4, 57.9]	[0.0, 52.2]	[0.0, 36.9]	[0.0, 45.9]	[0.0, 45.9]	[0.0, 10.4]
DCR	1 (33.3)	1 (25.0)	1 (25.0)	1 (25.0)	0	3 (42.9)	4 (80.0)	5 (62.5)	3 (50.0)	1 (16.7)	2 (39.2)
95% CI	[0.8, 90.6]	[0.6, 80.6]	[0.6, 80.6]	[0.6, 80.6]	[0.0, 60.2]	[9.9, 81.6]	[28.4, 99.5]	[24.5, 91.5]	[11.8, 88.2]	[0.4, 64.1]	[25.8, 53.9]

*BOR* best overall response, *CI* confidence interval, *DCR* disease control rate, *ORR* overall response rate, *PD* progressive disease, *PR* partial response, *Reg* regimen.

BOR was based on the investigator’s assessment of disease status using RECIST 1.1.

CR and PR were confirmed by repeat assessments performed not less than 4 weeks after the criteria for the response was first met.

The 95% CI was calculated using the exact (Clopper–Pearson) interval.

Reg 1: 3qw dosing, 4-week treatment cycle, continuous; Reg 3: 3qw dosing, 3-week cycle; 2 weeks on treatment and 1 week off treatment.

The best-percentage change from baseline by treatment group and mutation status is presented in Fig. 2b. Of note, nine patients included in the trial were found to have TP53 mutations after central p53 status reassessment using FoundationOne test (Fig. 2b). These patients were included in the study based on negative p53 mutation status assessed by local p53 testing (7 patients) or based on Amplichip p53 testing performed with a CRO (two patients). Six of these nine patients progressed rapidly after the study start.

### Pharmacokinetics and pharmacodynamics

Oral treatment with CGM097 in the fasted state resulted in a median time to reach maximum plasma concentrations (T_max_) ranging from 1.6 to 4.17 h. Within the dose range of 10–700 mg, mean plasma exposures (area under the curve [AUC_last_] and maximum concentration [C_max_]) increased with increasing dose with no major deviation from dose proportionality, after single and repeated doses across all dose levels. Inter-patient variability expressed as a coefficient of variation was low to moderate for AUC_last_ and C_max_ (12–63.3%, refer to Supplementary Table S1).

p53 pathway activation by CGM097 was demonstrated in patients by induction of its downstream target GDF-15 in serum (Fig. 2c). Increases in GDF-15 (fold change from baseline) were correlated with both CGM097 exposure and C_max_ (*P* value = 0.004 for both).

### Pharmacokinetic and pharmacodynamic modelling

**Fig. 3:**
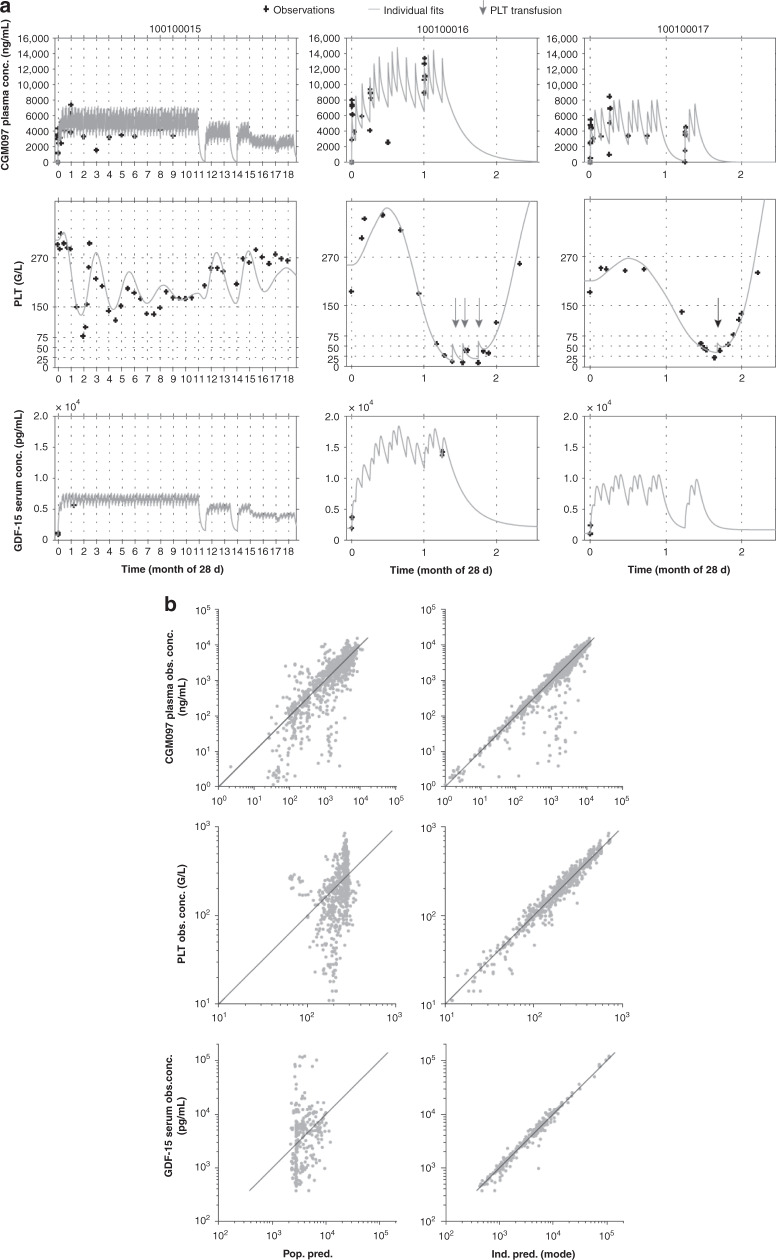
Pharmacokinetic and pharmacodynamic modelling of CGM097 concentrations, platelets count and GDF-15 kinetics. **a** Representative time course of the individual model fitted predictions versus observations for PK, PLT and GDF-15. Each vertical panel corresponds to a separate patient. Top, middle and bottom panels correspond, respectively, to individual CGM097 plasma concentrations (ng/mL), PLT counts (G/L) and GDF-15 serum concentrations (pg/mL) versus time profiles (day). Observed data are represented by black crosses and individual model fits by a continuous grey line. For the PLT time course, transfusion events are represented by vertical arrows. **b** Observed versus individual and population predicted for PK, PLT and GDF-15. The left and right panels represent observed versus predicted plots at the population level and individual level, respectively. The top, middle and bottom panels represent observed versus predicted plots for CGM097 plasma concentrations (ng/mL), PLT counts (G/L) and GDF-15 serum concentrations (pg/mL), respectively. conc concentration, d day, GDF-15 growth differentiation factor-15, Ind. pred. individual predicted, Obs. observed, PK phamacokinetic, PLT platelets, Pop. pred. population predicted. Dots represent the observed data.

The established clinical PK/PD models appropriately described the PK, and the temporal relationship of drug action on platelets, and serum GDF-15. Matched individual observed vs. predicted time course for PK, platelets, and GDF-15, as well as goodness-of-fit plots, are shown in Fig. 3a and b, respectively. PK, platelet, and GDF-15 time course and variability parameters were estimated with good precision (Supplementary Table S2).

The PK of CGM097 was best described by a two-compartment model with a delayed first-order absorption (parameters *Tlag and ka*) and a linear elimination. The model was parameterised by apparent oral clearance (*CL/F*), apparent central volume of distribution (*Vc/F*), apparent inter-compartmental clearance (*Q/F*), and apparent peripheral volume of distribution (*Vp/F*). Population PK parameter estimates are reported in Supplementary Table S2. Population values were estimated to be 1.62 L/h for CL/F and 67 L for Vc/F (inter-patient variability: CL/F, 70%; Vc/F, 35%). The absorption rate constant (*ka*) and absorption lag time (*Tlag*) were estimated to be 1.42 1/h and 0.39 h, respectively.

To better understand CGM097-induced thrombocytopenia profiles, a semi-mechanistic myelosuppression model of platelet kinetics reproducing the hematopoietic maturation process was established by linking CGM097 plasma concentrations with observed blood platelet profiles. This model was modified from Friberg et al.^14^ Drug central compartment PK model concentrations *[C]* were assumed to induce death of early proliferative hematopoietic cells via both a direct and an indirect drug effect through an effect compartment (Supplementary Fig. S6). The parameter estimates (PD platelet) are reported in Supplementary Table S2. Effect on early proliferative hematopoietic cells was associated with a mean maturation time (MMT) of ~264 h. Large IIV of 314 (CV%) was estimated on the early cell killing parameter, defined by the term *slPi*, reflecting the broad sensitivity across patients in response to bone marrow toxicity. All parameters were identifiable with relative standard errors below 30% and a low residual error (0.152), indicating that the model was able to adequately describe the population trends for long-term platelet profiles up to 20 months.

To characterise CGM097 exposure effect on GDF-15 kinetics and potential correlation with exposure effect on thrombocytopenia, a GDF-15 PK/PD model was developed. GDF-15 kinetics was best described by an indirect response model (Type III) with a drug effect on the zero-order input rate of GDF-15 production (*kin*). Baseline conditions were estimated independently of production and turnover (*kout*). The parameter estimates (PD GDF-15) are reported in Supplementary Table S2. All parameters were identifiable with relative standard errors below 20% and a low residual error (0.191). The population value for GDF-15 at baseline (*gdfZ*) was estimated at 2780 pg/mL with a large IIV (CV% = 121). Intermittent 2 weeks on/1 week off dosing resulted in a return of GDF-15 level to baseline prior to the next treatment cycle. The correlation between model-derived individual drug effect on GDF-15 production (denoted by *slGi)* against the individual drug effect on immature hematopoietic cells (denoted by *slPi)* is illustrated in Supplementary Fig. S8. These data, derived from 45 patients, with an *R*^2^ of 0.51, suggest that GDF-15 induction could be utilised as a prospective marker in the identification of patients at higher risk of developing delayed thrombocytopenia.

## Discussion

CGM097 administration in MDM2-amplified tumour models resulted in upregulation of p53 target genes, such as CDKN1A, BBC3, GDF-15 and HDM2 at the mRNA level, as previously published. In addition, preclinical efficacy experiments demonstrated that CGM097 could induce tumour regressions with daily dosing similar and consistent with other p53/MDM2 inhibitors^5,20–22^ However, here we report that similar preclinical regressions could be reached with an alternative dosing regimen, especially with the 3qw regimen, which may have the best therapeutic index based on the low weekly dose and C_ave__-__ss_ compared with the daily treatment regimen, and the low C_max-ss_ compared with the 2qw and 3 days on/4 days off regimens.

These preclinical in vivo studies supported clinical exploration while providing preliminary evidence for dosing regimens. In the first-in-human phase I study of CGM097, adult patients with selected advanced solid tumours received different doses of CGM097 via two different dosing regimens: a 3qw continuous regimen and an alternative 3qw, 2 weeks on and 1 week off regimen, to allow bone marrow recovery. Clinical benefit was observed in 20 out of 51 patients. Of these, 19 patients had SD and a melanoma patient treated with CGM097 at 300 mg (3qw dosing, continuous) achieved PR. No CR was reported. Of the patients with mutated p53 included in the trial (Fig. 2b), two-thirds progressed rapidly after the study start. In most of the enrolled patients, p53 sequencing was also reassessed using next-generation sequencing (NGS) with FoundationOne test,^23^ where the mutations in 9 patients were discovered. There are several potential reasons for these discrepancies in p53 sequencing. First, they could be due to the sensitivity and ability of different assays to detect mutations with lower allelic frequency. SNaPshot,^24–26^ Sanger^27,28^ and sequencing using AmpliChip Kit^29^ (with limit of detection ranging from ~10–20%) were utilised for sequencing prior to patient enrollment in the study, while NGS FoundationOne test (with a limit of detection of 5%) was used retrospectively. The latter, in addition to higher sensitivity, offers improved range and depth of coverage. Second, a minimum sequencing of exons 5–8 in TP53 was required prior to enrollment, with the rationale that over 90% of mutations in p53 are found in the DNA-binding domain located between those exons and therefore mutations outside of these would be rare.^30^ For FoundationOne, NGS sequencing reportable range encompasses all exons, and potentially provides another source for the discrepancy. Lastly, and perhaps the most likely reason for discrepancies in p53 mutation detection is the fact that sequencing was done on different biopsy samples. Tumour biopsies could be collected from different locations. In addition, archival tissue collected months before the study enrollment was allowed for screening. As the incidence of p53 mutations may increase with therapy-related genomic instability, above reasons may have contributed to the detection of p53 mutations after the patient has been enrolled in the study.^31^

A major concern with p53-reactivating therapies is its effect on normal cells, which includes the stabilisation of p53 resulting in increased apoptosis in these cells. In a clinical trial of RG7112 in liposarcoma, the most common toxicity was haematological, with 30% of patients experiencing grade 4 neutropenia, and 15% experiencing prolonged grade 4 thrombocytopenia.^32–34^ However, whether the haematologic toxicity correlated with prior exposure to genotoxic therapies is unknown. There were also reports of an increased incidence of p53 mutations following prolonged Nutlin-3a exposure^35^ and concerns about this effect on the development of new cancers.^36^ In this study, the most frequently reported treatment-related AEs included thrombocytopenia, nausea, leukopenia, vomiting, and fatigue, and the most common treatment-related grade 3 or 4 AEs were thrombocytopenia, lymphopenia and neutropenia. No DLTs were observed in the range of tested doses within the DLT period of 4 weeks, as the haematological-related events were generally observed after day 28 (cycle 1) due to the delayed onset of myelosuppression relative to the start of drug treatment. The most frequently occurring treatment-related grade ≥3 AE was thrombocytopenia. Since HDM2 plays a role in normal hematopoiesis, this effect was considered as on-target and consistent with other HDM2-antagonist agents.^32–34^

MTD was not reached. The study was halted prematurely due to a strategic decision by Novartis to develop HDM201, a second-generation HDM2 inhibitor. In comparison to CGM097, HDM201 demonstrates improved preclinical properties. Even though both HDM201 and CGM097 are highly potent and selective inhibitors of HDM2-p53 interaction, with IC50 values of 0.2 nM versus 1.5 nM, respectively, HDM201 showed improved physicochemical properties^2^ and an improved PK profile allowing continuous low-dose and pulse high-dose dosing regimen.^18^ Therefore, after a thorough comparison of the preclinical and clinical data from both HDM2 inhibitor programmes, Novartis decided to prioritise the clinical development of HDM201.

The ability to quantitatively assess drug-induced haematological toxicity is of great value for dose optimisation, administration schedules, and in predicting complications for patients who undergo prolonged periods of myelosuppression. Here, we developed a semi-mechanistic thrombocytopenia model for CGM097, and showed that the model can adequately describe the relationship between drug concentrations and the long-term time course of platelets following the administration of CGM097. Appropriate description of platelet profiles required the model to have both a direct and an indirect drug-induced depletion of immature cells, and a drug effect on systemic regulation. The model was able to describe the longitudinal time course of platelet changes associated with up to 20 months of treatment duration. Clinically, one of the major toxicities associated with CGM097 administration was found to be the delayed onset of thrombocytopenia. The delayed onset of CGM097-induced thrombocytopenia and the long platelet recovery time were addressed through an adapted dosing strategy and guided by the thrombocytopenia model, including higher dose treatment followed by a drug holiday period to maintain the total dose per cycle. Switching to an intermittent dosing schedule resulted in a manageable platelet profile while still achieving a similar average dose per cycle compared to a continuous schedule. This is reflected by the relatively long model-derived estimate for MMT on circulating platelets of ~264 h. Unlike previously reported shorter MMT values for classic cytotoxic chemotherapy agents,^14^ the estimated MMT value in this study was consistent with that of other HDM2 inhibitors and therefore likely to be a common characteristic of HDM2 inhibitors.^37^ In addition, large variability in developing CGM097-induced thrombocytopenia was observed among patients. These clinical effects were further reflected by the very large model-derived inter-individual variability of the drug thrombocytopenia potency parameter at ~314%. Indeed, the ability to predict the likelihood that a patient will develop delayed, drug-induced thrombocytopenia early enough would allow proactive toxicity management of the patient. The PK/PD model-derived individual drug potency on GDF-15 production on day 1 was associated with the drug thrombocytopenia potency, raising the possibility to use GDF-15 induction as a prospective marker in the identification of patients at a higher risk of developing delayed thrombocytopenia (Supplementary Fig. S1). The link of circulating GDF-15 as an early predictive marker of delayed thrombocytopenia related to CGM097 or other HDM2 inhibitors would require further investigations beyond the exploratory analysis of this study. Overall, this work provides an integrated quantitative understanding of thrombocytopenia and the GDF-15 biomarker changes in response to HDM2 inhibitors, with potential use in dosing regimen optimisation and patient benefit.

While CGM097 did not show a clinically significant number of tumour regressions, more patients rather experienced disease stabilisations, we were however able to reactivate p53 at therapeutically relevant doses. This study provides a relevant reference for potential drug combination studies that may hold greater promise for MDM2 inhibitors than monotherapies. This study further confirms that for p53-inducing drugs, the therapeutic window, especially in the context of delayed haematological toxicity, may require highly specific scheduling as well as predictive PD markers to improve patient benefit while mitigating the safety risk of severe thrombocytopenia. In conclusion, although this study was prematurely discontinued, we have developed the methods to improve the dosing of MDM2 inhibitor using extensive PK/PD modelling. The important learnings derived from the CGM097X2101 first-in-human study are supporting the new generation MDM2 inhibitors in development which are currently tested in clinics.

## Supplementary information

CGM097 Manuscript Supplementary Material

Prof. Demetri - Change of authorship request form

Dr Tan - Change of authorship request form

Dr. Van Bree - Change of authorship request form

Dr. Ferretti - Change of authorship request form

Dr. Bauer - Change of authorship request form

Dr. Halilovic - Change of authorship request form

Dr. Fabre and Dr. Cassier - Change of authorship request form

Dr. Guerreiro - - Change of authorship request form

Dr. Hourcade-Potelleret - Change of authorship request form

Dr. Jeay - Change of authorship request form

Dr. Jullion - Change of authorship request form

Dr. Meille and Dr. Dummer - Change of authorship request form

Dr. Wuerthner - Change of authorship request form

## Data Availability

Novartis will not provide access to patient-level data if there is a reasonable likelihood that individual patients could be re-identified. Phase 1 studies, by their nature, present a high risk of patient re-identification; therefore, patient individual results for phase 1 studies cannot be shared. In addition, clinical data, in some cases, have been collected subject to contractual or consent provisions that prohibit transfer to third parties. Such restrictions may preclude granting access under these provisions. Where co-development agreements or other legal restrictions prevent companies from sharing particular data, companies will work with qualified requestors to provide summary information where possible.
