# Supplementary material for: Pharmacokinetic–pharmacodynamic guided optimisation of dose and schedule of CGM097, an HDM2 inhibitor, in preclinical and clinical studies
Source: Br J Cancer. 2021 Jun 17;125(5):687–98. doi: 10.1038/s41416-021-01444-4 (PMC8405607; doi:10.1038/s41416-021-01444-4)
Supplement: Supplementary file 1 — CGM097 Manuscript Supplementary Material [file 41416_2021_1444_MOESM1_ESM.docx]

**Supplementary Table S1:** Summary of primary PK parameters for CGM097 by time point and treatment

| **Treatment** | **Visit** | | **Statistics** | **AUClast (h*ng/mL)** | **Cmax (ng/mL)** | | | **Tmax (hour)** |
| --- | --- | --- | --- | --- | --- | --- | --- | --- |
| 10 mg Reg.1 | Cycle 1 Day 1 | | n | 3 | 3 | | | 3 |
|  |  | | Mean (SD) | 1739.93 (711.538) | 162.23 (76.945) | | |  |
|  |  | | CV% mean | 40.9 | 47.4 | | |  |
|  | Cycle 1 Day 8 | | n | 3 | 3 | | | 3 |
|  |  | | Mean (SD) | 749.13 (139.257) | 135.00 (26.230) | | |  |
|  |  | | CV% mean | 18.6 | 19.4 | | |  |
|  | Cycle 2 Day 1 | | n | 3 | 3 | | | 3 |
|  |  | | Mean (SD) | 855.06 (178.234) | 150.67 (28.042) | | |  |
|  |  | | CV% mean | 20.8 | 18.6 | | |  |
| 20 mg Reg.1 | Cycle 1 Day 1 | | n | 4 | 4 | | | 4 |
|  |  | | Mean (SD) | 3635.22 (1088.174) | 226.25 (75.057) | | |  |
|  |  | | CV% mean | 29.9 | 33.2 | | |  |
|  | Cycle 1 Day 8 | | n | 3 | 4 | | | 4 |
|  |  | | Mean (SD) | 1900.63 (701.167) | 292.50 (97.647) | | |  |
|  |  | | CV% mean | 36.9 | 33.4 | | |  |
|  | Cycle 2 Day 1 | | n | 2 | 2 | | | 2 |
|  |  | | Mean (SD) | 2474.20 (1289.411) | 417.00 (209.304) | | |  |
|  |  | | CV% mean | 52.1 | 50.2 | | |  |
| 40 mg Reg.1 | Cycle 1 Day 1 | | n | 4 | 4 | | | 4 |
|  |  | | Mean (SD) | 8642.17 (3823.696) | 648.25 (230.749) | | |  |
|  |  | | CV% mean | 44.2 | 35.6 | | |  |
|  | Cycle 1 Day 8 | | n | 1 | 2 | | | 2 |
|  |  | | Mean (SD) | 4557.75 | 775.50 (4.950) | | |  |
|  |  | | CV% mean |  | 0.6 | | |  |
|  | Cycle 2 Day 1 | | n | 3 | 3 | | | 3 |
|  |  | | Mean (SD) | 4412.87 (549.163) | 779.67 (162.346) | | |  |
|  |  | | CV% mean | 12.4 | 20.8 | | |  |
| 80 mg Reg.1 | Cycle 1 Day 1 | | n | 4 | 4 | | | 4 |
|  |  | | Mean (SD) | 25777.65 (9327.937) | 1705.00 (286.880) | | |  |
|  |  | | CV% mean | 36.2 | 16.8 | | |  |
|  | Cycle 1 Day 8 | | n | 4 | 4 | | | 4 |
|  |  | | Mean (SD) | 12555.36 (5037.766) | 1992.50 (761.331) | | |  |
|  |  | | CV% mean | 40.1 | 38.2 | | |  |
|  | Cycle 2 Day 1 | | n | 3 | 3 | | | 3 |
|  |  | | Mean (SD) | 13763.53 (6414.576) | 2150.00 (972.780) | | |  |
|  |  | | CV% mean | 46.6 | 45.2 | | |  |
| 150 mg Reg.1 | Cycle 1 Day 1 | | n | 4 | 4 | | | 4 |
|  |  | | Mean (SD) | 28371.87 (10386.60) | 2170.75 (822.210) | | |  |
|  |  | | CV% mean | 36.6 | 37.9 | | |  |
|  | Cycle 1 Day 8 | | n | 3 | 3 | | | 3 |
|  |  | | Mean (SD) | 19288.47 (2468.197) | 3280.00 (537.308) | | |  |
|  |  | | CV% mean | 12.8 | 16.4 | | |  |
|  | Cycle 2 Day 1 | | n | 4 | 4 | | | 4 |
|  |  | | Mean (SD) | 18402.22 (7266.522) | 2845.00 (1103.766) | | |  |
|  |  | | CV% mean | 39.5 | 38.8 | | |  |
| 300 mg Reg.1 | Cycle 1 Day 1 | | n | 7 | 7 | | | 7 |
|  |  | | Mean (SD) | 76083.41 (30334.21) | 5641.43 (2310.667) | | |  |
|  |  | | CV% mean | 39.9 | 41.0 | | |  |
|  | Cycle 1 Day 8 | | n | 5 | 5 | | | 5 |
|  |  | | Mean (SD) | 48412.97 (23680.85) | 7566.00 (3552.363) | | |  |
|  |  | | CV% mean | 48.9 | 47.0 | | |  |
|  | Cycle 2 Day 1 | | n | 4 | 4 | | | 4 |
|  |  | | Mean (SD) | 52368.63 (33042.78) | 8020.00 (5077.611) | | |  |
|  |  | | CV% mean | 63.1 | 63.3 | | |  |
| 400 mg Reg.1 | Cycle 1 Day 1 | | n | 4 | 5 | | | 5 |
|  |  | | Mean (SD) | 83589.63 (25096.84) | 4714.00 (1055.405) | | |  |
|  |  | | CV% mean | 30.0 | 22.4 | | |  |
|  | Cycle 1 Day 8 | | n | 3 | 3 | | | 3 |
|  |  | | Mean (SD) | 42948.14 (6165.260) | 6700.00 (1670.898) | | |  |
|  |  | | CV% mean | 14.4 | 24.9 | | |  |
|  | Cycle 2 Day 1 | | n | 5 | 5 | | | 5 |
|  |  | | Mean (SD) | 46810.46 (11680.57) | 6962.00 (1506.659) | | |  |
|  |  | | CV% mean | 25.0 | 21.6 | | |  |
| 300 mg Reg.3 | Cycle 1 Day 1 | | n | 6 | | 6 | | 6 |
|  |  | | Mean (SD) | 44312.60 (6454.001) | | 3061.67 (824.995) | |  |
|  |  | | CV% mean | 14.6 | | 26.9 | |  |
|  | Cycle 1 Day 8 | | n | 6 | | 6 | | 6 |
|  |  | | Mean (SD) | 24549.59 (6984.906) | | 3910.00 (1201.383) | |  |
|  |  | | CV% mean | 28.5 | | 30.7 | |  |
|  | Cycle 1 Day 12 | | n | 6 | | 6 | | 6 |
|  |  | | Mean (SD) | 165660.7 (27956.75) | | 4385.00 (866.920) | |  |
|  |  | | CV% mean | 16.9 | | 19.8 | |  |
|  | Cycle 2 Day 1 | | n | 4 | | 5 | | 5 |
|  |  | | Mean (SD) | 16358.31 (1460.291) | | 2706.00 (433.855) | |  |
|  |  | | CV% mean | 8.9 | | 16.0 | |  |
| 500 mg Reg.3 | | Cycle 1 Day 1 | n | 6 | | | 6 | 6 |
|  | |  | Mean (SD) | 76082.40 (28847.89) | | | 4641.67 (1726.516) |  |
|  | |  | CV% mean | 37.9 | | | 37.2 |  |
|  | | Cycle 1 Day 8 | n | 4 | | | 6 | 6 |
|  | |  | Mean (SD) | 41750.26 (14158.86) | | | 7538.33 (2451.990) |  |
|  | |  | CV% mean | 33.9 | | | 32.5 |  |
|  | | Cycle 1 Day 12 | n | 5 | | | 5 | 5 |
|  | |  | Mean (SD) | 502939.9 (197712.9) | | | 10700.00 (4454.958) |  |
|  | |  | CV% mean | 39.3 | | | 41.6 |  |
|  | | Cycle 2 Day 1 | n | 6 | | | 6 | 6 |
|  | |  | Mean (SD) | 26839.03 (8596.600) | | | 4808.33 (1606.069) |  |
|  | |  | CV% mean | 32.0 | | | 33.4 |  |
| 700 mg Reg.3 | | Cycle 1 Day 1 | n | 5 | | | 5 | 5 |
|  | |  | Mean (SD) | 79130.82 (9456.635) | | | 5586.00 (985.713) |  |
|  | |  | CV% mean | 12.0 | | | 17.6 |  |
|  | | Cycle 1 Day 8 | n | 4 | | | 4 | 4 |
|  | |  | Mean (SD) | 58196.53 (8550.117) | | | 9572.50 (1988.121) |  |
|  | |  | CV% mean | 14.7 | | | 20.8 |  |
|  | | Cycle 1 Day 12 | n | 2 | | | 3 | 3 |
|  | |  | Mean (SD) | 236590.1 (183561.3) | | | 9236.67 (5443.550) |  |
|  | |  | CV% mean | 77.6 | | | 58.9 |  |
|  | | Cycle 2 Day 1 | n | 4 | | | 4 | 4 |
|  | |  | Mean (SD) | 35842.49 (9334.222) | | | 5862.50 (732.911) |  |
|  | |  | CV% mean | 26.0 | | | 12.5 |  |

Reg 1: 3qw dosing, 4-week treatment cycle, continuous; Reg 3: 3qw dosing, 3-week cycle; 2 weeks on treatment and 1 week off treatment.

AUC_last_, area under the curve from time = 0 to last measurable concentration, T_last_=24h on cycle 1day 1 and T_last_=8h on cycle 1 day 8 and cycle 2 day 1; C_max_, maximum plasma concentration; CV, coefficient of variation; SD, standard deviation; t_max_, time to reach C_max_.

**Supplementary Table S2. Model-derived parameter estimates (RSE%) for PK, platelet, and GDF-15**

| Model | Parameter | Unit | Estimate | RSE% | CV IIV | RSE% | Parameter description |
| --- | --- | --- | --- | --- | --- | --- | --- |
| **PK** | *Ka* | *h^-1^* | 2.05 | 17 | 98.7 | 12 | First-order absorption rate constant |
|  | *Tlag* | *h* | 0.383 | 4 | 21.9 | 17 | Absorption lag-time |
|  | *V1/F* | *L* | 92.6 | 6 | 33.7 | 13 | Apparent central volume of distribution |
|  | *β V_tBWkg* | *-* | 1.12 | 20 |  |  | Body weight on central volume of distribution |
|  | *CL/F* | *L/h* | 1.62 | 10 | 67 | 11 | Apparent clearance |
|  | *Q/F* | *L/h* | 2.81 | 31 | 50 | 70 | Apparent Inter-compartmental clearance |
|  | *V2/F* | *L* | 7.11 | 50 | 200 | 14 | Apparent peripheral volume of distribution |
|  | σ_add_ | *ng/mL* | 1 ^a^ | - |  |  | Residual additive error |
|  | σ_prop_ | *-* | 0.389 | 3 |  |  | Residual proportional error |
|  |  |  |  |  |  |  |  |
| **PD Platelet** | *PLTz* | *G/L* | 276 | 6 | 40.9 | 11 | Baseline platelet |
|  | *MMT* | *h* | 264 | 13 | 35 ^a^ | - | Mean maturation time of drug affected cells |
|  | *Alp* | *G/L* | 27.8 | 27 | 70 ^a^ | - | Platelet transfusion dose |
|  | *ke0* | *h^-1^* | 3.29E-08 | 12 | 30 ^a^ | - | Effect compartment rate |
|  | *SlPd* | *mL/ng* | 3.93E-05 | 9 | 30 ^a^ | - | Drug direct thrombocytopenia potency |
|  | *SlPi* | *mL/ng* | 0.0176 ^a^ | - | 314 | 17 | Drug indirect thrombocytopenia potency through effect compartment |
|  | *SEP* | *-* | 0.74 | 0.46 | -^b^ | - | Indirect effect thrombocytopenia potency power |
|  | *Spw* | *-* | 0.595 | 7 | 20 ^a^ | - | Systemic regulation |
|  | *cfr* | *mL/ng* | −1.62 | 20 | 100 ^a^ | - | Drug effect potency on systemic regulation |
|  | *lPW* | *-* | 0.146 | 6 | 20 ^a^ | - | Local regulation |
|  | σ_add_ | *G/L* | 5 ^a^ | - |  |  | Residual additive error |
|  | σ_prop_ | *-* | 0.152 | 4 |  |  | Residual proportional error |
|  |  |  |  |  |  |  |  |
| **PD**  **GDF-15** | *Sg* | *mL/ng* | 0.00078 | 12 | 33.4 ^a^ | - | Drug potency |
|  | *kout* | *h^-1^* | 0.08 | 18 | 90.7 | 14 | Turnover rate |
|  | *gdfZ* | *pg/mL* | 2.78E+03 | 19 | 121 | 11 | Baseline concentration |
|  | *kin* | *pg/h* | 175 | 15 | 63.9 | 17 | Production rate |
|  | σ_add_ | *pg/mL* | 0.0118 ^a^ | - |  |  | Residual additive error |
|  | σ_prop_ | *-* | 0.191 | 6 |  |  | Residual proportional error |

Parameters are presented as the estimate followed by the relative standard error (RSE%) in parentheses.

CV IIV, inter-individual variability as coefficient of variation.

σ_add_, additive residual variability; σ_prop_, proportional residual variability. The residual variability (for modeling of PK, PLT and GDF-1) was modeled using a combined error model (proportional and additive).

^a^ Denotes parameter estimates that were fixed and therefore no relative standard error is reported.

^b^ parameter was fixed to 0.

**Supplementary Figure S1.**

1. **Schematic representation of the model used to describe the pharmacokinetics of CGM097.** *ka*: first-order absorption rate constant; *Tlag*: absorption lag time; *V1*: apparent volume of distribution in central compartment; *V2*: apparent volume of distribution in peripheral compartment; *CL*: apparent clearance; *C*: plasma concentration time course of CGM097; *Q*: apparent inter-compartmental clearance.


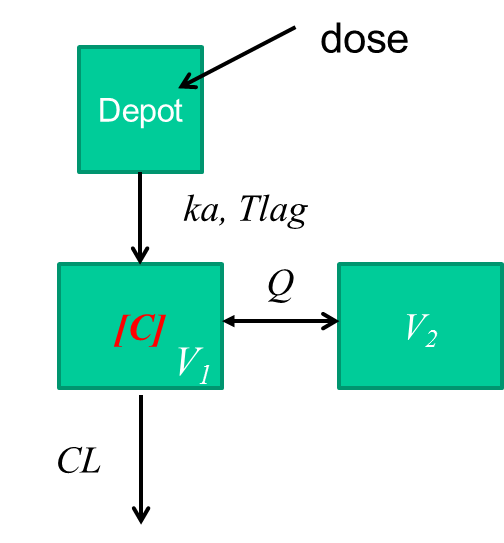


1. **Schematic representation of the semi-physiological model mimicking the hematopoietic cell maturation and regulation of platelets (PLT).** A self-renewing progenitor compartment ($P1)$is connected to transit compartments ($P2, P3, P4)$ representing maturation process, and leading to a circulating blood compartment ($P5$). A systemic regulation mechanism ($sFBK)$ mimics the increase in proliferation rate as PLT count decreases below baseline, while a local feedback ($lFBK$) mechanism mimics how the number of cells in the first compartment affects the proliferation of cells in subsequent compartments. This model includes a drug effect decoupled from systemic feedback. Drug effect involves both a direct ($slpd\cdot C$) and an indirect $(slPi\cdot E^{SEP})$diminution of the proliferation ability of progenitor cells, as well as an indirect effect on the systemic feedback through an effect compartment. PLT transfusion events ($u\left( t \right))$ are considered as a PLT transfusion dose in the circulating blood compartment.


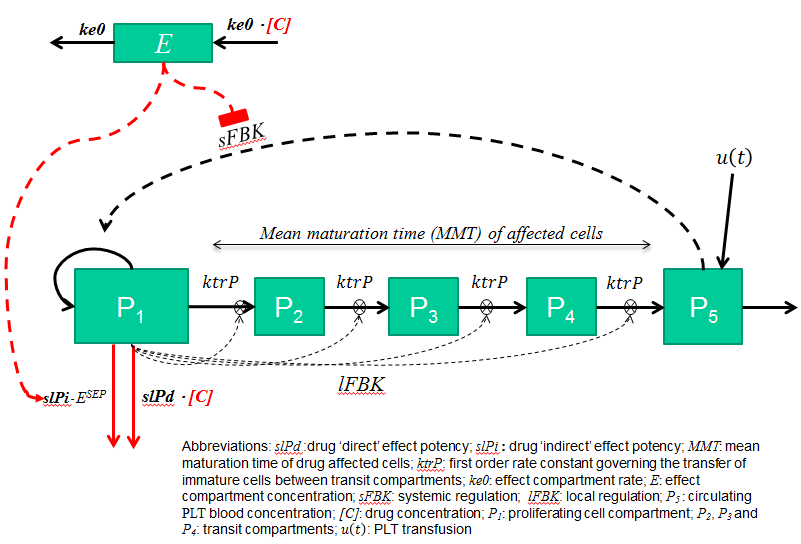


**c) Schematic representation of the PK/PD model for GDF-15**. An indirect response PD model with drug stimulation of GDF-15 production was used to describe the kinetics of GDF-15 in serum. Baseline conditions were estimated independently of production and turnover (non-stationary initial conditions).

[C]: CGM097 plasma concentration


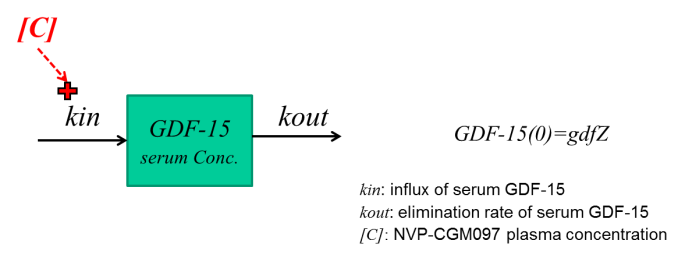


**Supplementary Figure S2.** Standard Goodness-of-fit diagnostics obtained with the PK model. Top panel: CWRES (Conditional Weighted Residuals) vs. time (left) and NPDE (Normalized Prediction Distribution Errors) vs. time (right). Bottom panel: CWRES vs. predicted concentrations (IPRED) (left) and NPDE vs. IPRED (right). Observed data are represented with dots.


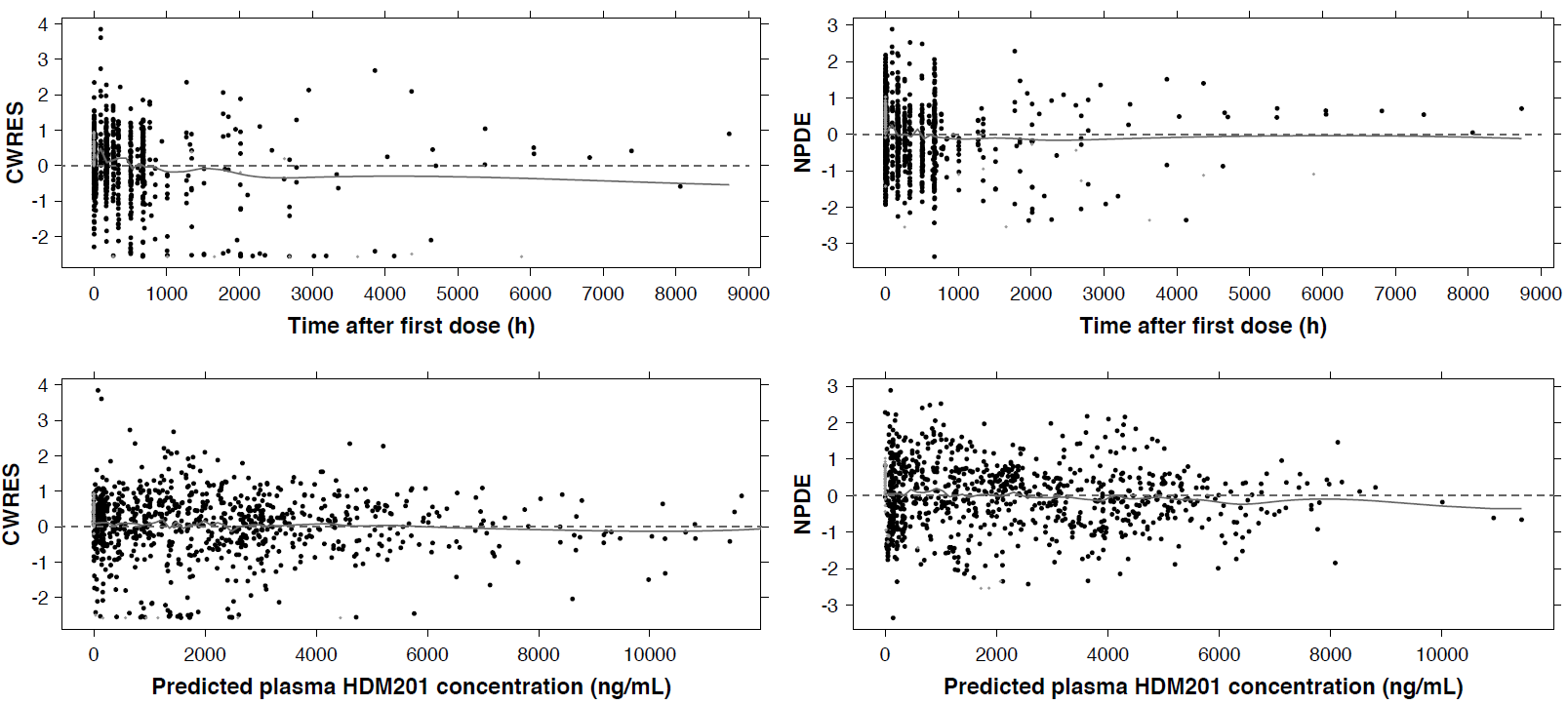


**Supplementary Figure S3.** Standard Goodness-of-fit diagnostics obtained with the PK/PD PLT model. Top panel: CWRES vs. time (left) and NPDE vs. time (right). Bottom panel: CWRES vs. predicted concentrations (IPRED) (left) and NPDE vs. IPRED (right). Observed data are represented with dots.


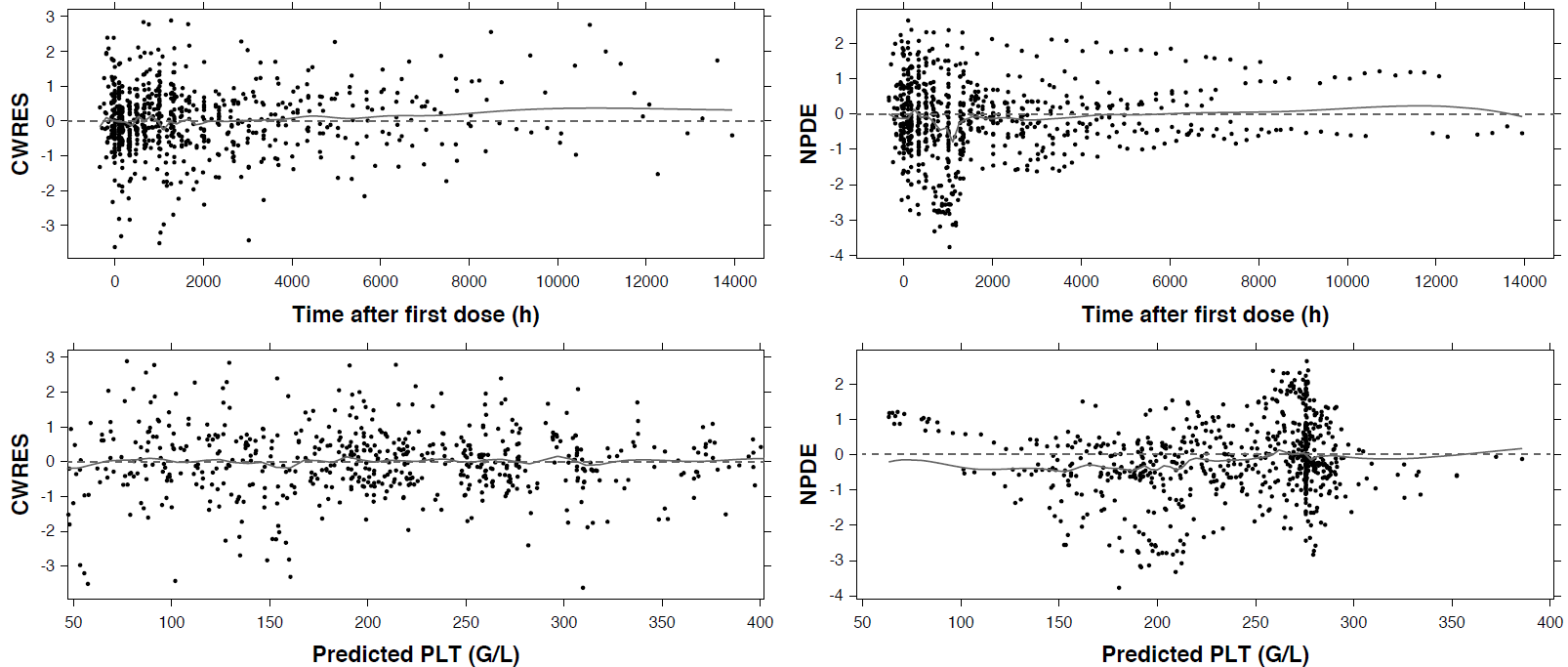


**Supplementary Figure S4.** Standard Goodness-of-fit diagnostics obtained with the PK/PD GDF 15 model. Top panel: CWRES vs. time (left) and NPDE vs. time (right). Bottom panel: CWRES vs. predicted concentrations (IPRED) (left) and NPDE vs. IPRED (right). Observed data are represented with dots.


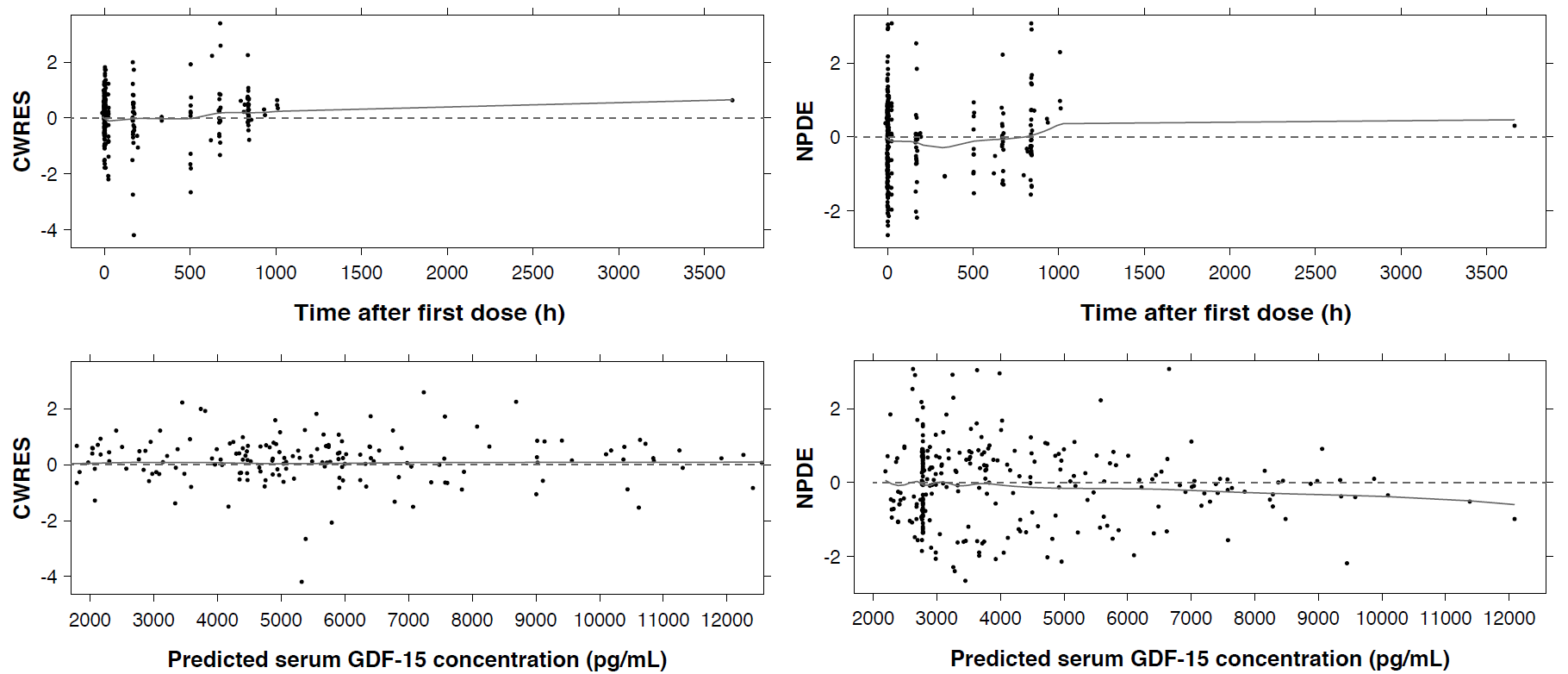


**Supplementary Figure S5.** Visual predictive check of PK (A), PLT (B) and GDF-15 (C). Lines correspond to 10^th^, 50^th^, and 90^th^ percentiles of the observed data. The light grey-shaded area represent the 90% confidence interval for the 50^th^ percentile of the predicted data, whereas the upper and lower dark-shaded areas represent the 90% confidence intervals for the 90^th^ and 10^th^ percentile of the predicted data.
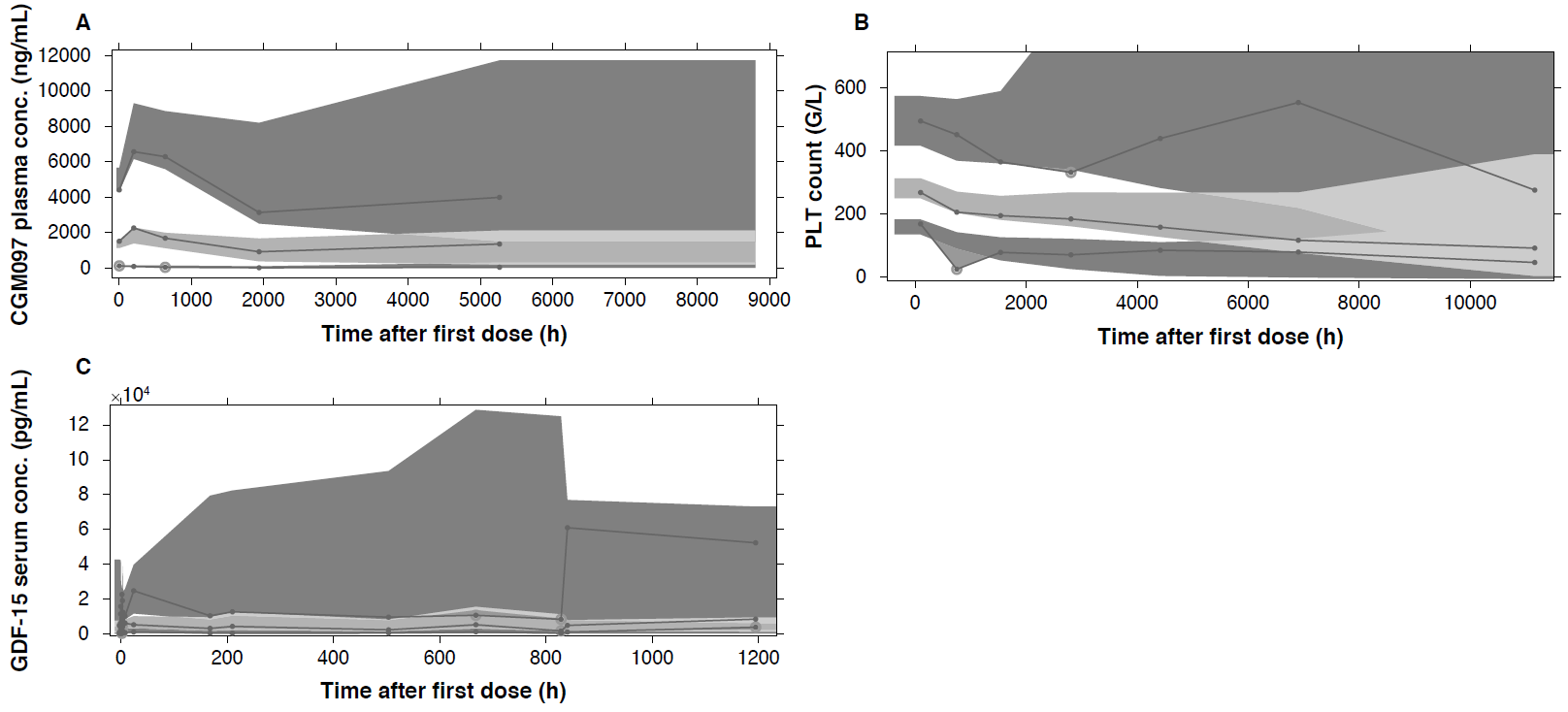


**Supplementary Figure S6.**

Efficacy in liposarcoma (LPS) xenograft models. Daily treatment with CGM097 dose-dependently induced tumor regression in MDM2-amplified 778 well differentiated (WDLPS) and LP6 dedifferentiated liposarsoma (DDLPS) models.
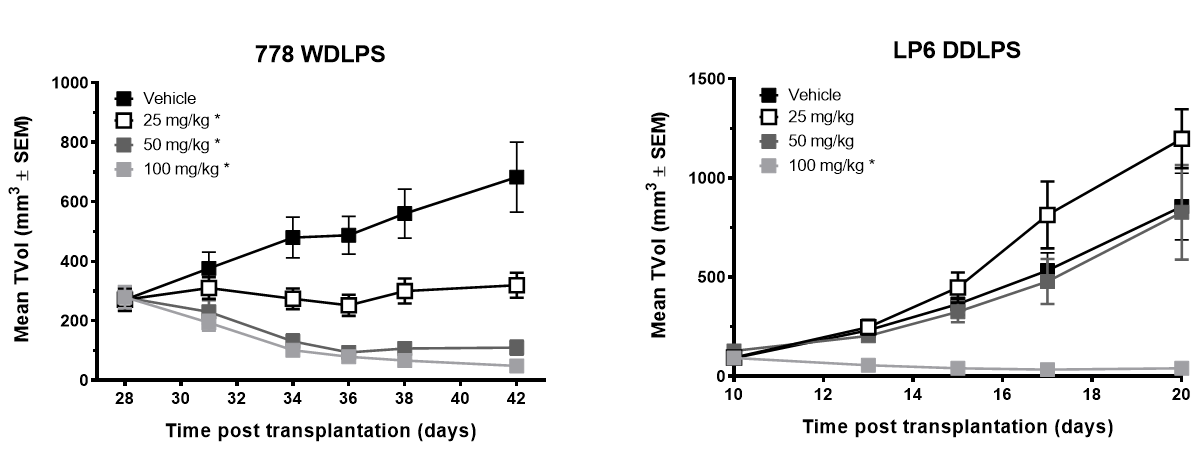


**Supplementary Figure S7.** CGM097 average dose per day by cohort. As no obvious schedule dependency was observed for preclinical efficacy (based on total dose), dose intensity $AvDose$ was calculated to allow comparison between dosing regimens 1 and 3. Dose intensity was derived using the following formula:$AvDose=\frac{d\cdot n_{adm}}{Tcycle}$ where $d$ is the planned dose, $n_{adm}$ the total number of administrations during the cycle and $Tcycle$ the cycle duration in days.
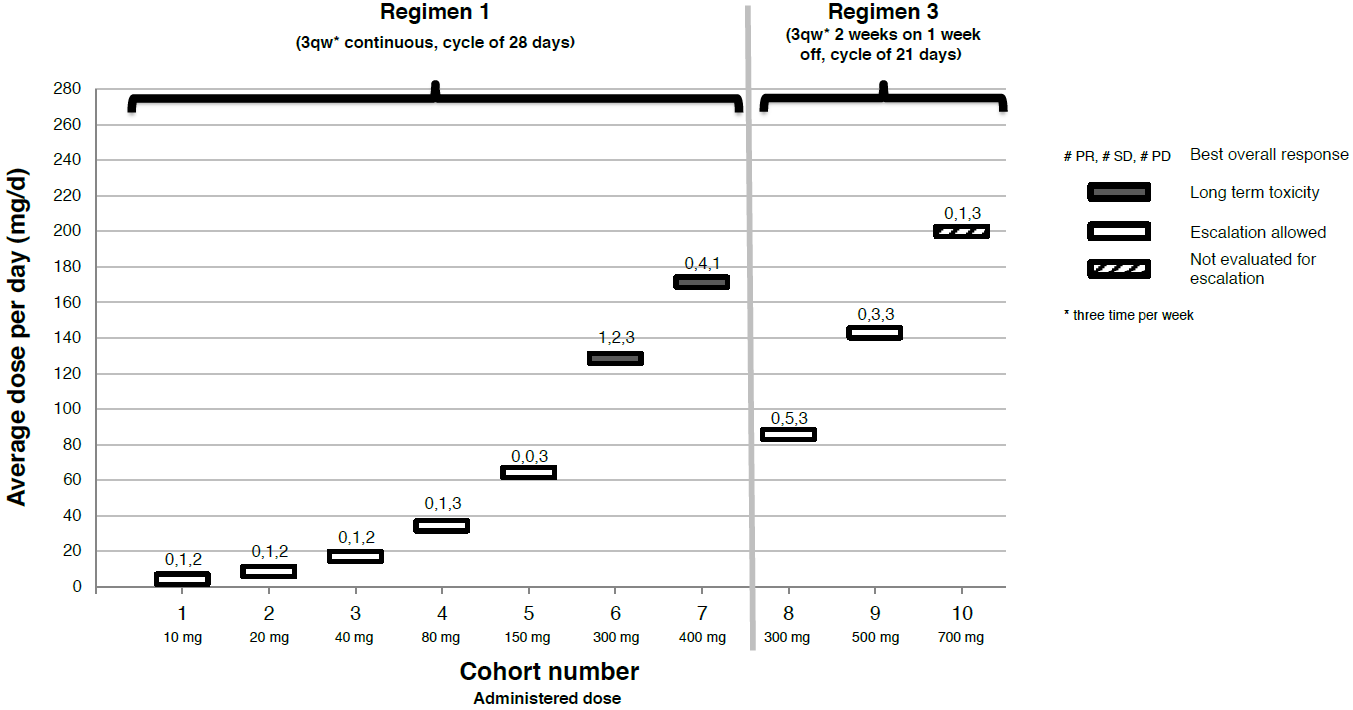


**Supplementary Figure S8.** Analysis exploring the link between individual drug potency on GDF15 (*slGi*) production and the individual drug potency on immature hematopoietic cells (*slPi*). Paired data derived from 45 patients.


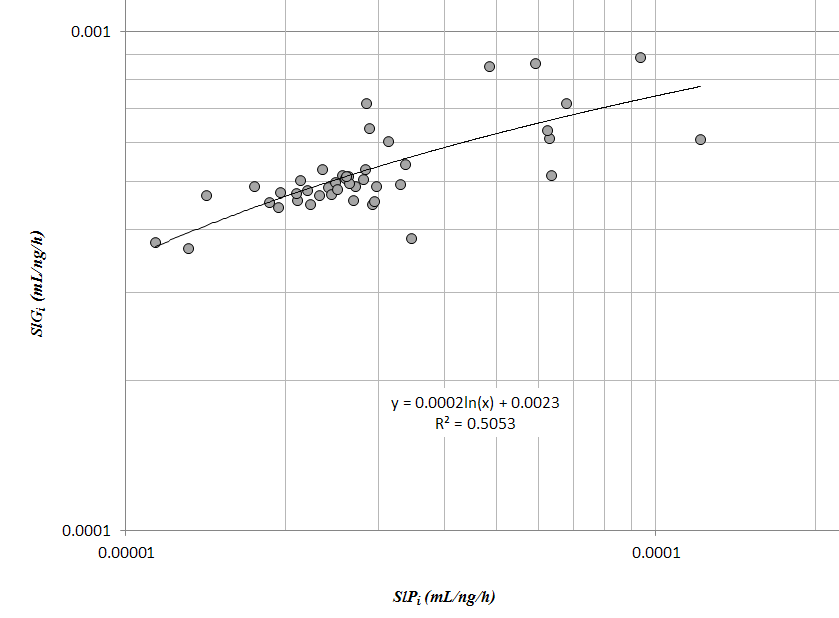


**Supplementary Data: The following equations describe the PK, PLT, and GDF-15 model**

**PK**

$$\begin{matrix} ke=\frac{Cl}{V} \\ k12=\frac{Q}{V} \\ k21=Q/V2 \end{matrix}$$

$$\begin{matrix} \frac{dQg}{dt}=-ka\cdot Qg(t-\tau) \\ \frac{dQc}{dt}=ka\cdot Qg(t-\tau)-ke\cdot Qc-k12\cdot Qc+k21\cdot Qp \\ \frac{dQp}{dt}=k12\cdot Qc-k21\cdot Qp \\ C=Qc/V \end{matrix}$$

**PLT**

$$\begin{matrix} ktrP=\frac{4}{MMTP} \\ phiP=ktrP \\ EP=slPi\cdot E^{SEP}+slpd\cdot C \end{matrix}$$

$$\begin{matrix} sFBK=\left( \frac{PLTz}{P5} \right)^{sPW\cdot e^{cfr\cdot E}} \\ lFBK=({PLTz\cdot\frac{phiP}{ktrP}/P1)}^{lPW} \\ icP=PLTz\cdot\frac{phiP}{ktrP} \\ For the i^{th} patient having the j^{th} transfusion event of duration TINFP at time {TI}_{i,j}: \\ \left\{ \begin{aligned} {TI}_{i,j}\leq t\leq{TI}_{i,j}+TINFP u\left( t \right)=\frac{\alpha}{TINFP} with TINFP=0.5h \\ else u\left( t \right)=0 \end{aligned} \right. \end{matrix}$$

$$\begin{matrix} \frac{dP1}{dt}=ktrP\cdot\left( sFBK-EP \right)\cdot P1-ktrP\cdot P1 & P1\left( 0 \right)=icP \\ \frac{dP2}{dt}=ktrP\cdot lFBK\cdot P1-ktrP\cdot P2 & P2\left( 0 \right)=icP \\ \frac{dP3}{dt}=ktrP\cdot lFBK\cdot P2-ktrP\cdot P3 & P3\left( 0 \right)=icP \end{matrix}$$

$$\begin{matrix} \frac{dP4}{dt}=ktrP\cdot lFBK\cdot P3-ktrP\cdot P4 & P4\left( 0 \right)=icP \\ \frac{dP5}{dt}=ktrP\cdot lFBK\cdot P4-phiP\cdot P5+u(t) & P5\left( 0 \right)=PLTz \\ \frac{dE}{dt}=ke0\cdot C-ke0\cdot E & E\left( 0 \right)=0 \end{matrix}$$

**GDF-15**

$$Egdf=Sg\cdot C$$

$$\frac{dgdf}{dt}=kin\cdot\left( 1+Egdf \right) -koutg\cdot gdf gdf\left( 0 \right)=gdfz$$
