# Supplementary material for: Pharmacokinetic–pharmacodynamic guided optimisation of dose and schedule of CGM097, an HDM2 inhibitor, in preclinical and clinical studies
Source: Br J Cancer. 2021 Jun 17;125(5):687–98. doi: 10.1038/s41416-021-01444-4 (PMC8405607; doi:10.1038/s41416-021-01444-4)
Supplement: Supplementary file 3 — Dr Tan - Change of authorship request form [file 41416_2021_1444_MOESM3_ESM.pdf]

Change of Authorship Request Form

Important Information - Please Read

- This form should be used by authors to request any change in authorship (swapping/adding/deleting authors), including changes in corresponding authors.
- Please fully complete all sections. Use black ink and block capitals and provide each author's full name with the given name first followed by the family name.
- By signing this declaration, all authors guarantee that the order of the authors are in accordance with their scientific contribution, if applicable as different conventions apply per discipline, and that only authors have been added who made a meaningful contribution to the work.
- Please note, in author collaborations where there is formal agreement for representing the collaboration, it is sufficient for the representative or legal guarantor (usually the corresponding author) to complete and sign the Authorship Change Form on behalf of all authors next to the added/removed author(s). (Complete Section 3, followed by Section 6.)
- In author collaborations where there is no formal agreement for representing the collaboration and there are more than 10 authors, one may sign for all, provided the signer appends correspondence that attests that each of the authors have agreed to the change and the added/removed authors sign the form. (Complete Section 3, followed by Section 6.)
- Please note, we cannot investigate or mediate any authorship disputes. If you are unable to obtain agreement from all authors (including those who you wish to be removed) you must refer the matter to your institution(s) for investigation. Please inform us if you need to do this.
- Incomplete forms will be rejected.
- Please upload this form, fully completed, to the online submission system. We will consider the information you have provided to decide whether to approve the proposed change in authorship. We may decide to contact your institution for more information or undertake a further investigation, if appropriate, before making a final decision.

Please fill out the below sections in full:

Section 1

Manuscript number:	CL-2020-7444R1
Manuscript title:	Pharmacokinetic -pharmacodynamic guided optimization of dose and schedule of CGM097, and HDM2 inhibitor, in preclinical and clinical studies

Section 2

Please provide the previous authorship, in the order shown on the manuscript before the changes were introduced. Please indicate the corresponding author by adding (CA) behind the name:

	First Name(s)	Family Name(s)
1st Author	SEBASTIAN (CA)	BAUER
2nd Author	GEORGE D.	DEMETRI
3rd Author	SEBASTIEN	JEAY
4th Author	REINHARD	DUMMER
5th Author	NELSON	GUERREIRO
6th Author	DANIEL	TAN
7th Author	ASTRID	JULLION
8th Author	CHRISTOPHE	MEILLE
9th Author	STEPHANE	FERRETTI
10th Author	LAURENCE	VAN BREE

Please use additional page for more than 10 authors.

Section 3

Please use this section to explain your reasons for changing the authorship of your manuscript, e.g. what necessitated the change in authorship. You may also use this section to detail changes to "contributed equally" statements, etc.

The authorship of the manuscript was updated based on the contributions made by some coauthors during the revision process to enable the final submission of the manuscript.

Section 4

Proposed new authorship. Please provide your new authorship list in the order you would like it to appear on the manuscript. Please indicate the corresponding author by adding (CA) behind the name. If the Corresponding Author has changed, please indicate the reason under section 3.

	First Name(s)	Family Name(s) (this name will appear in full on the final publication and will be searchable in various abstract and indexing databases)	Affiliated Institute	Date
1st Author	SEBASTIAN (CA)	BAUER	Department of Medical Oncology, Sarcoma Center, West German Cancer Center, University of Duisburg-Essen, Germany and DKTK Partner Site Essen	8th April, 2021
2nd Author	GEORGE D.	DEMETRI	Dana-Farber Cancer Institute and Ludwig Center at Harvard Medical School, Boston, Massachusetts, USA	8th April, 2021
3rd Author	ENSAR	HALILOVIC	Novartis Institutes for BioMedical Research (NIBR), Cambridge, Massachusetts, USA	8th April, 2021
4th Author	REINHARD	DUMMER	University Hospital Zurich, Zurich, Switzerland	8th April, 2021

5th Author	CHRISTOPHE	MEILLE	Novartis Institutes for BioMedical Research (NIBR), Basel, Switzerland	8th April, 2021
6th Author	DANIEL	TAN	National Cancer Center Singapore, Singapore	8th April, 2021
7th Author	NELSON	GUERREIRO	Novartis Institutes for BioMedical Research (NIBR), Basel, Switzerland Current address: F. Hoffmann-La Roche AG, Grenzacherstrasse 124, Basel, Switzerland	8th April, 2021
8th Author	ASTRID	JULLION	Novartis Institutes for BioMedical Research (NIBR), Basel, Switzerland	8th April, 2021
9th Author	STEPHANE	FERRETTI	Novartis Institutes for BioMedical Research (NIBR), Basel, Switzerland	8th April, 2021
10th Author	SEBASTIEN	JEAY	Novartis Institutes for BioMedical Research (NIBR), Basel, Switzerland Current address: Idorsia Pharmaceuticals Ltd, Allschwil, Switzerland	8th April, 2021

Please use additional page for more than 10 authors.

Section 5

Author contribution, Acknowledgement and Disclosures. Please use this section to provide a new disclosure statement and, if appropriate, acknowledge any contributors who have been removed as authors and ensure you state what contribution any new authors made (if applicable per the journal or book (series) policy). Please ensure these are updated in your manuscript - after approval of the change(s) - as our production department will not transfer the information in this form to your manuscript.

New acknowledgements:	Not applicable

New Disclosures (financial and non-financial interests, funding):	Not applicable
New Author Contributions statement (if applicable per the journal policy):	Not applicable

State 'Not applicable' if there are no new authors.

Section 6

Declaration of agreement. All authors, unchanged, new and removed must sign this declaration.
(NB: Please print the form, (docu)-sign and return/upload a scanned copy. Please note that signatures that have been inserted as an image file are acceptable as long as it is handwritten. Typed names in the signature box are unacceptable.)
*Please delete as appropriate. Delete all of the bold if you were on the original authorship list and are remaining as an author.

	First Name(s)	Family Name(s)		Signature	Date
1st Author	SEBASTIAN	BAUER	I agree to the proposed new authorship (change in order) shown in section 4		
2nd Author	GEORGE D.	DEMETRI	I agree to the proposed new authorship (change in order) shown in section 4		
3rd Author	ENSAR	HALILOVIC	I agree to the proposed new authorship (change in order) shown in section 4		

4th Author	REINHARD	DUMMER	I agree to the proposed new authorship (change in order) shown in section 4		
5th Author	CHRISTOPHE	MEILLE	I agree to the proposed new authorship (change in order) shown in section 4		
6th Author	DANIEL	TAN	I agree to the proposed new authorship (change in order) shown in section 4		16.4.21
7th Author	NELSON	GUERREIRO	I agree to the proposed new authorship (change in order) shown in section 4		
8th Author	ASTRID	JULLION	I agree to the proposed new authorship (change in order) shown in section 4		
9th Author	STEPHANE	FERRETTI	I agree to the proposed new authorship (change in order) shown in section 4		
10th Author	SEBASTIEN	JEAY	I agree to the proposed new authorship (change in order) shown in section 4		

Please use an additional sheet if there are more than 10 authors.

In case of author collaborations with formal agreement:

	Name of consortium/consortia	First name(s)	Family name(s)		Signature	Date
Representative /Legal guarantor				I agree to the proposed new authorship shown in section 4 /and the addition/removal* of my name to the authorship list /and the proposed change in corresponding author		

Both added/removed authors should complete the information in the first table under Section 6.

Additional Page for proposed author changes (Section 2):

Author number	First Name(s)	Family Name(s)
11th Author	ENSAR	HALILOVIC
12th Author	FLORENCE	HOURCADE-POTELLERET
13th Author	CLAIRE	FABRE
14th Author	JENS U.	WUERTHNER
15th Author	PHILIPPE A.	CASSIER

Additional page for agreement to author changes (Section 4):

Author Number	First Name(s)	Family Name(s) (this name will appear in full on the final publication and will be searchable in various abstract and indexing databases)	Affiliated Institute	Date
11th Author	LAURENCE	VAN BREE	Novartis Institutes for BioMedical Research (NIBR), Basel, Switzerland	8th April, 2021
12th Author	FLORENCE	HOURCADE-POTELLERET	Novartis Institutes for BioMedical Research (NIBR), Basel, Switzerland	8th April, 2021
13th Author	JENS U.	WUERTHNER	Novartis Institutes for BioMedical Research (NIBR), Basel, Switzerland<br>Current address: ADC Therapeutics, Route de la Corniche 3B, 1066 Epalinges, Switzerland	8th April, 2021
14th Author	CLAIRE	FABRE	Novartis Institutes for BioMedical Research (NIBR), Basel, Switzerland	8th April, 2021
15th Author	PHILIPPE A.	CASSIER	Department of Medical Oncology, Centre Léon Bérard, Lyon, France	8th April, 2021

Additional page for agreement to author changes (Section 6):

Author Number	First Name(s)	Family Name(s)		Signature	Date
11th Author	LAURENCE	VAN BREE	I agree to the proposed new authorship (change in order) shown in section 4		
12th Author	FLORENCE	HOURCADE-POTELLERET	I agree to the proposed new authorship (change in order) shown in section 4		
13th Author	JENS U.	WUERTHNER	I agree to the proposed new authorship (change in order) shown in section 4		
14th Author	CLAIRE	FABRE	I agree to the proposed new authorship (change in order) shown in section 4		
15th Author	PHILIPPE A.	CASSIER	I agree to the proposed new authorship (change in order) shown in section 4		
			I agree to the proposed new authorship shown in section 4 /and the addition/removal* of my name to the authorship list /and the proposed change in corresponding author		
			I agree to the proposed new authorship shown in section 4 /and the addition/removal* of my name to the authorship list /and the proposed change in corresponding author		

			I agree to the proposed new authorship shown in section 4 /and the addition/removal* of my name to the authorship list /and the proposed change in corresponding author		
			I agree to the proposed new authorship shown in section 4 /and the addition/removal* of my name to the authorship list /and the proposed change in corresponding author		
			I agree to the proposed new authorship shown in section 4 /and the addition/removal* of my name to the authorship list /and the proposed change in corresponding author		
			I agree to the proposed new authorship shown in section 4 /and the addition/removal* of my name to the authorship list /and the proposed change in corresponding author		
			I agree to the proposed new authorship shown in section 4 /and the addition/removal* of my name to the authorship list /and the proposed change in corresponding author		
			I agree to the proposed new authorship shown in section 4 /and the addition/removal* of my name to the authorship list /and the proposed change in corresponding author		
			I agree to the proposed new authorship shown in section 4 /and the addition/removal* of my name to the authorship list /and the proposed change in corresponding author		
			I agree to the proposed new authorship shown in section 4 /and the addition/removal* of my name to the authorship list /and the proposed change in corresponding author		
			I agree to the proposed new authorship shown in section 4 /and the addition/removal* of my name to the authorship list /and the proposed change in corresponding author		
			I agree to the proposed new authorship shown in section 4 /and the addition/removal* of my name to the authorship list /and the proposed change in corresponding author		
			I agree to the proposed new authorship shown in section 4 /and the addition/removal* of my name to the authorship list /and the proposed change in corresponding author		

			I agree to the proposed new authorship shown in section 4 /and the addition/removal* of my name to the authorship list /and the proposed change in corresponding author		
