# Supplementary material for: Pharmacokinetic–pharmacodynamic guided optimisation of dose and schedule of CGM097, an HDM2 inhibitor, in preclinical and clinical studies
Source: Br J Cancer. 2021 Jun 17;125(5):687–98. doi: 10.1038/s41416-021-01444-4 (PMC8405607; doi:10.1038/s41416-021-01444-4)
Supplement: Supplementary file 6 — Dr. Bauer - Change of authorship request form [file 41416_2021_1444_MOESM6_ESM.pdf]

New Disclosures (financial and non-financial interests, funding):	Not applicable
New Author Contributions statement (if applicable per the journal policy):	Not applicable

State 'Not applicable' if there are no new authors.

Section 6

Declaration of agreement. All authors, unchanged, new and removed must sign this declaration.

(NB: Please print the form, (docu)-sign and return/upload a scanned copy. Please note that signatures that have been inserted as an image file are acceptable as long as it is handwritten. Typed names in the signature box are unacceptable.)

*Please delete as appropriate. Delete all of the bold if you were on the original authorship list and are remaining as an author.

	First Name(s)	Family Name(s)		Signature	Date
1st Author	SEBASTIAN	BAUER	I agree to the proposed new authorship (change in order) shown in section 4		12.APR2021
2nd Author	GEORGE D.	DEMETRI	I agree to the proposed new authorship (change in order) shown in section 4		
3rd Author	ENSAR	HALILOVIC	I agree to the proposed new authorship (change in order) shown in section 4		
