# Supplementary material for: Pharmacokinetic–pharmacodynamic guided optimisation of dose and schedule of CGM097, an HDM2 inhibitor, in preclinical and clinical studies
Source: Br J Cancer. 2021 Jun 17;125(5):687–98. doi: 10.1038/s41416-021-01444-4 (PMC8405607; doi:10.1038/s41416-021-01444-4)
Supplement: Supplementary file 8 — Dr. Fabre and Dr. Cassier - Change of authorship request form [file 41416_2021_1444_MOESM8_ESM.pdf]


Additional page for agreement to author changes (Section 6):

Author Number	First Name(s)	Family Name(s)		Signature	Date
11th Author	LAURENCE	VAN BREE	I agree to the proposed new authorship (change in order) shown in section 4		
12th Author	FLORENCE	HOURCADE-POTELLERET	I agree to the proposed new authorship (change in order) shown in section 4		
13th Author	JENS U.	WUERTHNER	I agree to the proposed new authorship (change in order) shown in section 4		
14th Author	CLAIRE	FABRE	I agree to the proposed new authorship (change in order) shown in section 4		14/04/2021
15th Author	PHILIPPE A.	CASSIER	I agree to the proposed new authorship (change in order) shown in section 4		14-APR-21
