# Supplementary material for: Pharmacokinetic–pharmacodynamic guided optimisation of dose and schedule of CGM097, an HDM2 inhibitor, in preclinical and clinical studies
Source: Br J Cancer. 2021 Jun 17;125(5):687–98. doi: 10.1038/s41416-021-01444-4 (PMC8405607; doi:10.1038/s41416-021-01444-4)
Supplement: Supplementary file 12 — Dr. Jullion - Change of authorship request form [file 41416_2021_1444_MOESM12_ESM.pdf]

4th Author	REINHARD	DUMMER	I agree to the proposed new authorship (change in order) shown in section 4		
5th Author	CHRISTOPHE	MEILLE	I agree to the proposed new authorship (change in order) shown in section 4		
6th Author	DANIEL	TAN	I agree to the proposed new authorship (change in order) shown in section 4		
7th Author	NELSON	GUERREIRO	I agree to the proposed new authorship (change in order) shown in section 4		
8th Author	ASTRID	JULLION	I agree to the proposed new authorship (change in order) shown in section 4	[signature]	15APR2021
9th Author	STEPHANE	FERRETTI	I agree to the proposed new authorship (change in order) shown in section 4		
10th Author	SEBASTIEN	JEAY	I agree to the proposed new authorship (change in order) shown in section 4		

Please use an additional sheet if there are more than 10 authors.

In case of author collaborations with formal agreement:

	Name of consortium/consortia	First name(s)	Family name(s)		Signature	Date
Representative /Legal guarantor				I agree to the proposed new authorship shown in section 4 /and the addition/removal* of my name to the authorship list /and the proposed change in corresponding author		

Both added/removed authors should complete the information in the first table under Section 6.
